# Gradients of PI(4,5)P_2_ and PI(3,5)P_2_ Jointly Participate in Shaping the Back State of *Dictyostelium* Cells

**DOI:** 10.3389/fcell.2022.835185

**Published:** 2022-02-04

**Authors:** Dong Li, Feifei Sun, Yihong Yang, Hui Tu, Huaqing Cai

**Affiliations:** ^1^ School of Life Sciences, University of Science and Technology of China, Hefei, China; ^2^ National Laboratory of Biomacromolecules, Institute of Biophysics, Chinese Academy of Sciences, Beijing, China; ^3^ College of Life Sciences, University of Chinese Academy of Sciences, Beijing, China

**Keywords:** polarity, back state, phosphoinositide signaling, *Dictyostelium*, migration

## Abstract

Polarity, which refers to the molecular or structural asymmetry in cells, is essential for diverse cellular functions. *Dictyostelium* has proven to be a valuable system for dissecting the molecular mechanisms of cell polarity. Previous studies in *Dictyostelium* have revealed a range of signaling and cytoskeletal proteins that function at the leading edge to promote pseudopod extension and migration. In contrast, how proteins are localized to the trailing edge is not well understood. By screening for asymmetrically localized proteins, we identified a novel trailing-edge protein we named Teep1. We show that a charged surface formed by two pleckstrin homology (PH) domains in Teep1 is necessary and sufficient for targeting it to the rear of cells. Combining biochemical and imaging analyses, we demonstrate that Teep1 interacts preferentially with PI(4,5)P_2_ and PI(3,5)P_2_ *in vitro* and simultaneous elimination of these lipid species in cells blocks the membrane association of Teep1. Furthermore, a leading-edge localized myotubularin phosphatase likely mediates the removal of PI(3,5)P_2_ from the front, as well as the formation of a back-to-front gradient of PI(3,5)P_2_. Together our data indicate that PI(4,5)P_2_ and PI(3,5)P_2_ on the plasma membrane jointly participate in shaping the back state of *Dictyostelium* cells.

## Introduction

Dynamic anterior-posterior polarity is a hallmark of eukaryotic motile cells. Cell polarity can be organized spontaneously or under the guidance of extracellular biochemical and mechanical cues (Goehring and Grill, 2013; Campanale et al., 2017). Study of cell migration in the model system *Dictyostelium discoideum* has provided important insights into the mechanisms underlying the establishment and maintenance of cell polarity (King and Insall, 2009; Devreotes et al., 2017). Moreover, many key signaling or cytoskeletal molecules involved in polarity regulation were originally discovered in *Dictyostelium* and later found to be conserved in higher eukaryotic cells.

In *Dictyostelium*, signaling and cytoskeletal components responsible for polarity regulation are often localized or activated specifically at the leading edge (front) or trailing edge (back) of migrating cells, creating functionally distinct ends that promote cell migration. Events that occur at the leading edge include the activation of several Ras and Rac family GTPases, activation of mTORC2 and its substrates of the Akt/PKB family kinases, accumulation of the class I PI3-kinases (PI3Ks) and their product PIP_3_, and recruitment of a number of regulators of actin polymerization, such as the Scar/WAVE and Arp2/3 complexes responsible for pseudopod projection (Funamoto et al., 2002; Sasaki et al., 2004; Kamimura et al., 2008; Cai et al., 2010; Charest et al., 2010; Ura et al., 2012; Veltman et al., 2016). Events that occur at the trailing edge include the recruitment of the PIP_3_ phosphatase Pten and the generation of formin and myosin II-dependent actin cortex, which is necessary for back retraction (Iijima and Devreotes, 2002; Levi et al., 2002; Ramalingam et al., 2015; Litschko et al., 2019).

The polarized activities of these signaling and cytoskeletal molecules have implications beyond cell migration. For example, during macropinocytosis, leading-edge molecules, such as PIP_3_ and activated Ras, decorate the forming macropinocytic cups, whereas trailing-edge molecules, such as Pten, are excluded from the cup areas but occupy the rest of the cell membrane (Parent et al., 1998; Hoeller et al., 2013; Junemann et al., 2016; Veltman et al., 2016; Buckley et al., 2020). During cytokinesis, leading-edge molecules localize to the poles, whereas trailing-edge molecules are restricted to the cleavage furrow (Faix et al., 2001; Janetopoulos and Devreotes, 2006; King et al., 2010; Kee et al., 2012). In response to global chemoattractant stimulation, leading-edge molecules transiently translocate to the cell periphery, whereas the trailing-edge molecules transiently fall off from the cell periphery and into the cytosol before returning to the cell periphery (Parent et al., 1998; Iijima and Devreotes, 2002; Sasaki et al., 2004; Sobczyk et al., 2014). The same complementary pattern is observed even when the actin cytoskeleton is disrupted by Latrunculin A (LatA). In LatA-treated cells, leading- and trailing-edge components distribute in the cytoplasm and on the plasma membrane and respond to stimulation by transiently relocalizing onto or off the plasma membrane, respectively (Janetopoulos et al., 2004; Xu et al., 2007; Swaney et al., 2015).

How different molecules implicated in the regulation of polarity achieve their characteristic distribution during diverse cellular activities remains to be fully elucidated. Previous studies have demonstrated an important role of PIP_3_ in determining the front state of cells (Funamoto et al., 2001; Huang et al., 2003). Local accumulation of PIP_3_ occurs via reciprocally distributed PI3Ks and Pten and is amplified through positive feedback loops involving PI3Ks, Ras and Rac proteins, and the actin network (Iijima and Devreotes, 2002; Weiner et al., 2002; Sasaki et al., 2007; Arai et al., 2010; Matsuoka and Ueda, 2018). PIP_3_ then serves as binding sites for a number of effectors, including pleckstrin homology (PH) domain-containing proteins, which regulate leading edge activities (Parent et al., 1998; Funamoto et al., 2001; Zhang et al., 2010; Chen et al., 2012). The diametrically opposed distribution of PI3Ks and Pten and the resulting PIP_3_ gradient manifest even in the presence of LatA. When LatA-treated cells are exposed to a chemoattractant gradient, PIP_3_ and Pten accumulate toward the high side of the gradient or away from it, respectively (Janetopoulos et al., 2004; Hoeller and Kay, 2007).

Compared to the well-characterized front state, the molecular definition of the back state remains obscure. PI(4,5)P_2_ has been found to accumulate at the back of migrating cells and the cleavage furrow (Wong et al., 2005; Janetopoulos and Devreotes, 2006; Keizer-Gunnink et al., 2007; Lokuta et al., 2007; Gerisch et al., 2011; Miao et al., 2019). In line with this, PI3Ks and PLC have been proposed to remove PI(4,5)P_2_ preferentially at the leading edge, whereas PI5K and Pten, which produce PI(4,5)P_2_, exhibit complementary patterns of localization (Funamoto et al., 2002; Iijima and Devreotes, 2002; Keizer-Gunnink et al., 2007; Miao et al., 2019). Furthermore, PI(4,5)P_2_ depletion has been demonstrated to trigger hyperactivation of cellular protrusions, consistent with its role in determining the back state (Miao et al., 2017). However, judging by the distribution of several PI(4,5)P_2_ sensors, gradients of PI(4,5)P_2_ are fairly modest in migrating cells (Gerisch et al., 2011; Matsuoka and Ueda, 2018), which suggests the existence of additional regulators of back activities. In addition to PI(4,5)P_2_, PI(3,4)P_2_ has been suggested to regulate back events, largely based on study of the trailing-edge protein CynA and its PH domain-containing region (Swaney et al., 2015). Evidence was provided for the existence of a mutually inhibitory feedback loop between Ras activities at the leading edge and PI(3,4)P_2_ (Li et al., 2018). However, it is not clear whether binding to PI(3,4)P_2_ is a general feature of trailing-edge proteins and whether PI(4,5)P_2_ and PI(3,4)P_2_ act independently or cooperatively.

Compared to the number of leading-edge proteins identified thus far, few proteins have been found at the trailing edge, and even fewer have been found to exhibit behavior similar to back proteins in the absence of an intact actin cytoskeleton (Iijima and Devreotes, 2002; Levi et al., 2002; Swaney et al., 2010; Swaney et al., 2015). This precludes a complete mechanistic understanding of the back state of cells. To gain further insights into how the back state is defined, we performed a microscopy-based screen in *Dictyostelium* for proteins that localize specifically to the trailing edge. We focused on PH domain-containing proteins because of their known functions in phosphoinositide signaling and polarity regulation. In particular, we selected PH domain-containing proteins predicted to be less likely to bind PIP_3_ by a recursive-learning algorithm (Park et al., 2008). Over 50 proteins of unknown function were tagged with GFP and examined for intracellular distribution (Supplementary Figure S1). This approach uncovered a novel back protein we named trailing edge enriched protein 1 (Teep1, gene ID DDB_G0277777). Characterization of the localization mechanism of Teep1 suggests the existence of a back-to-front gradient of PI(3,5)P_2_ on the plasma membrane, which acts together with PI(4,5)P_2_ to modulate the posterior accumulation of Teep1 and to shape the back state of cells.

## Results

### Teep1 is a Novel Trailing-Edge Protein

**FIGURE 1:**
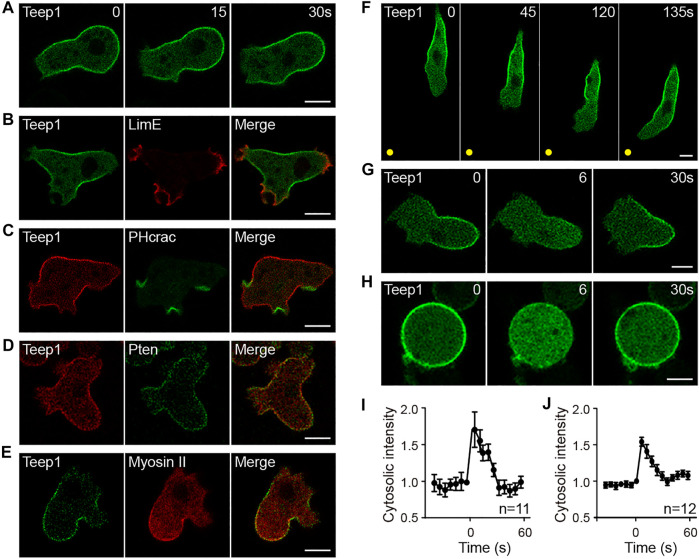
Teep1 is a novel trailing edge protein. **(A)** In randomly migrating cells, Teep1-GFP localizes at the trailing edge. **(B,C)** Teep1-GFP exhibits opposite distribution to LimE∆coil-RFP **(B)** and PHcrac-GFP **(C)**. **(D)** Colocalization of Teep1-RFP and Pten-GFP. **(E)** Colocalization of Teep1-GFP and myosin II-RFP. **(F)** Localization of Teep1-GFP in differentiated cells chemotaxing toward cAMP. The yellow dots mark the positions of the cAMP source. **(G)** Teep1-GFP translocates from the plasma membrane to the cytoplasm in response to cAMP stimulation (1 μM cAMP was added at time 0). **(H)** Teep1-GFP translocation in response to cAMP stimulation in the presence of 5 μM LatA. **(I,J)** Quantification of cAMP-induced Teep1-GFP translocation in the absence [**(I)**, mean ± SEM] or the presence [**(J)**, mean ± SEM] of LatA. Data was collected from three independent experiments. Scale bar, 5 μm.

We characterized the localization pattern of Teep1 in vegetative and differentiated cells. Vegetative cells produce distinctive leading-edge structures, macropinocytic cups and pseudopods, which drive bulk endocytosis and cell movement, respectively. Teep1-GFP was selectively excluded from these structures, resulting in an apparent back-to-front gradient in its plasma membrane association (Figure 1A; Supplementary Video S1). Colocalization with well-characterized marker proteins confirmed its trailing-edge enrichment. Teep1 exhibited opposite distribution to the leading edge-localized F-actin reporter LimEΔcoil and PIP_3_/PI(3,4)P_2_ reporter PHcrac (Figures 1B,C), whereas it largely colocalized with back proteins, including Pten and myosin II (Figures 1D,E). Consistent with the observations in vegetative cells, Teep1-GFP localized specifically to the side and rear of differentiated cells migrating along cAMP gradients (Figure 1F; Supplementary Video S2). Transient relocalization in response to global chemoattractant stimulation is another feature of back proteins. We observed that, upon the addition of cAMP to differentiated cells, Teep1-GFP translocated from the plasma membrane to the cytosol within 5–10 s and then returned to the plasma membrane in approximately 30 s (Figures 1G,I; Supplementary Video S3). A similar response was observed when vegetative cells were stimulated with folic acid (Supplementary Figure S2A). As reported for Pten (Iijima et al., 2004), the chemoattractant-induced translocation of Teep1 did not require an intact actin cytoskeleton (Figures 1H,J; Supplementary Video S4). Furthermore, when the LatA-treated cells were exposed to a cAMP gradient, Teep1 exhibited a complementary distribution to PHcrac, forming a crescent away from the higher concentration of cAMP (Supplementary Figure S2B). These experiments verified that Teep1 is a novel trailing-edge protein.

Several observations revealed that, among the previously characterized back proteins, including Pten, myosin II, PhdB (RG3, Rapgap3), and CynA (Moores et al., 1996; Iijima and Devreotes, 2002; Lee et al., 2014; Swaney et al., 2015), the localization pattern of Teep1 most resembles that of Pten. First, both Teep1 and Pten were absent from pseudopods and macropinocytic cups (Supplementary Figures S2C,D), whereas the PH domain of CynA and PhdB localized to the base of macropinocytic cups and newly formed macropinosomes, in addition to their posterior distribution (Supplementary Figures S2E,F) (Zhang et al., 2010; Li et al., 2018). Second, in response to stimulation, both Teep1 and Pten redistributed from the plasma membrane to the cytoplasm, and the response occurred with similar kinetics in the presence of LatA (Figures 1G,J) (Iijima et al., 2004). In contrast, the response of myosin II relied on an intact actin cytoskeleton (Levi et al., 2002). Third, in some cells co-expressing Teep1 and Pten, Teep1 appeared to be partially depleted from regions where Pten more strongly accumulated (Supplementary Figure S2G), indicating that the two proteins may share similar binding sites. Despite these similarities, Pten and Teep1 did not depend on each other for trailing-edge localization (Supplementary Figure S2H).

### The Pleckstrin Homology Domains of Teep1 Determine its Trailing Edge Localization

**FIGURE 2:**
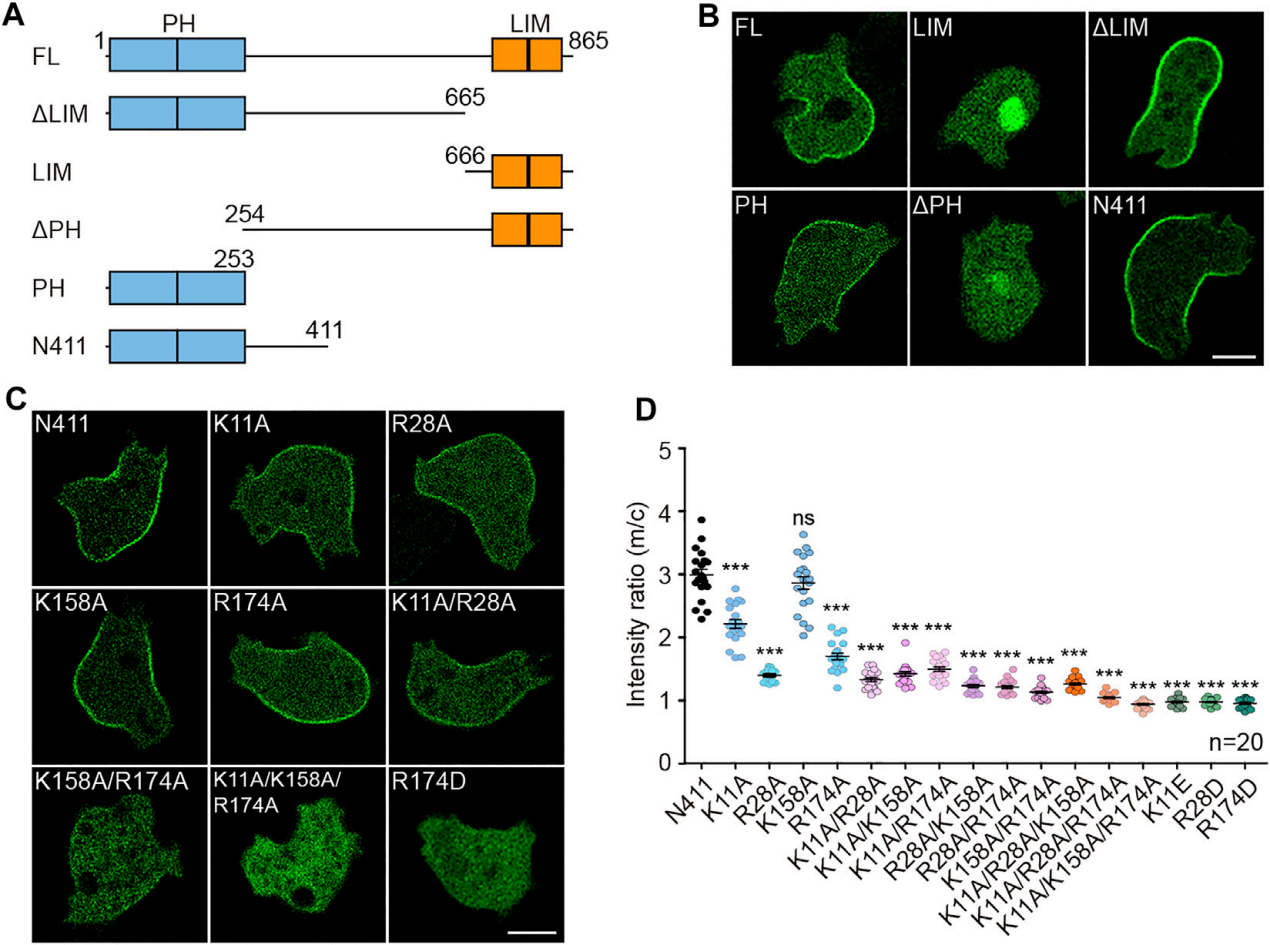
The PH domains of Teep1 determine its trailing-edge localization. **(A)** Schematic representation of Teep1 and the derived truncations. **(B)** Localization of GFP-tagged Teep1 and different truncations. **(C)** Localization of Teep1^N411^-GFP and Teep1^N411^-GFP bearing mutations in the PH domains. **(D)** Quantification of the membrane-to-cytosol fluorescent intensity ratios of Teep1^N411^-GFP and mutated Teep1^N411^-GFP. The scatter plot shows data points with means and SEM. Data was collected from at least two independent experiments. Scale bar, 5 μm.

To seek regions of Teep1 that regulate its trailing edge accumulation, we generated a series of GFP-tagged truncation constructs and examined their localization (Figures 2A,B). In addition to the two PH domains at the N-terminus, Teep1 contains two LIM domains at the C-terminus (Kadrmas and Beckerle, 2004). We found that the truncation containing LIM domains was located in the cytoplasm and nucleus. Deleting the LIM domains did not affect the localization of Teep1. Conversely, deleting the PH domains caused Teep1 to completely dissociate from the plasma membrane. The truncation containing only the PH domains was sufficient to drive the trailing-edge distribution, despite a lower expression level. Including an extension at the C-terminus, which possibly stabilized the PH domains, resulted in a truncated protein (Teep1^N411^) with an equivalent level of expression, extent of rear enrichment, and responsiveness to stimulation as the full-length protein (Figures 2A,B; Supplementary Figures S3A,B). Using this construct, we further examined the role of the PH domains in regulating the localization of Teep1.

Positively charged residues within the β1/β2 loop are often required for membrane-binding PH domains to interact with negatively charged lipids (Lemmon, 2008). Sequence alignment with the PH domain from PEPP1 (Dowler et al., 2000) revealed two conserved positively charged residues within each PH domain of Teep1 (Supplementary Figure S3C). We mutated these residues individually or in combination to either alanine or amino acid with an opposite charge (Figure 2C; Supplementary Figure S3E). The membrane-to-cytosol fluorescent intensity ratios were quantified following LatA treatment, which allowed easier assessment of the membrane-binding capacity of back proteins (Figure 2D) (Li et al., 2018; Matsuoka and Ueda, 2018). We found that single alanine mutations (except K158A) caused a partial reduction in the membrane association of Teep1^N411^, and incorporating additional alanine mutation further decreased the association. Teep1^N411^ completely lost membrane association when three residues were mutated to alanines simultaneously. In contrast, when these residues were switched to oppositely charged amino acids, a single mutation alone was sufficient to block the membrane localization (Figures 2C,D; Supplementary Figure S3E). In the protein homology/analogy recognition engine 2 (Phyre2) modeled structure, the four amino acids are positioned in two positively charged patches oriented to the same side, which could facilitate membrane targeting (Supplementary Figure S3D). These analyses indicate that the PH domains of Teep1 form a charged surface that is necessary and sufficient for sensing the properties of the plasma membrane that determine the back state.

### PI(3,4)P_2_ or PI(4,5)P_2_ Gradient is not Sufficient for Targeting Teep1

**FIGURE 3:**
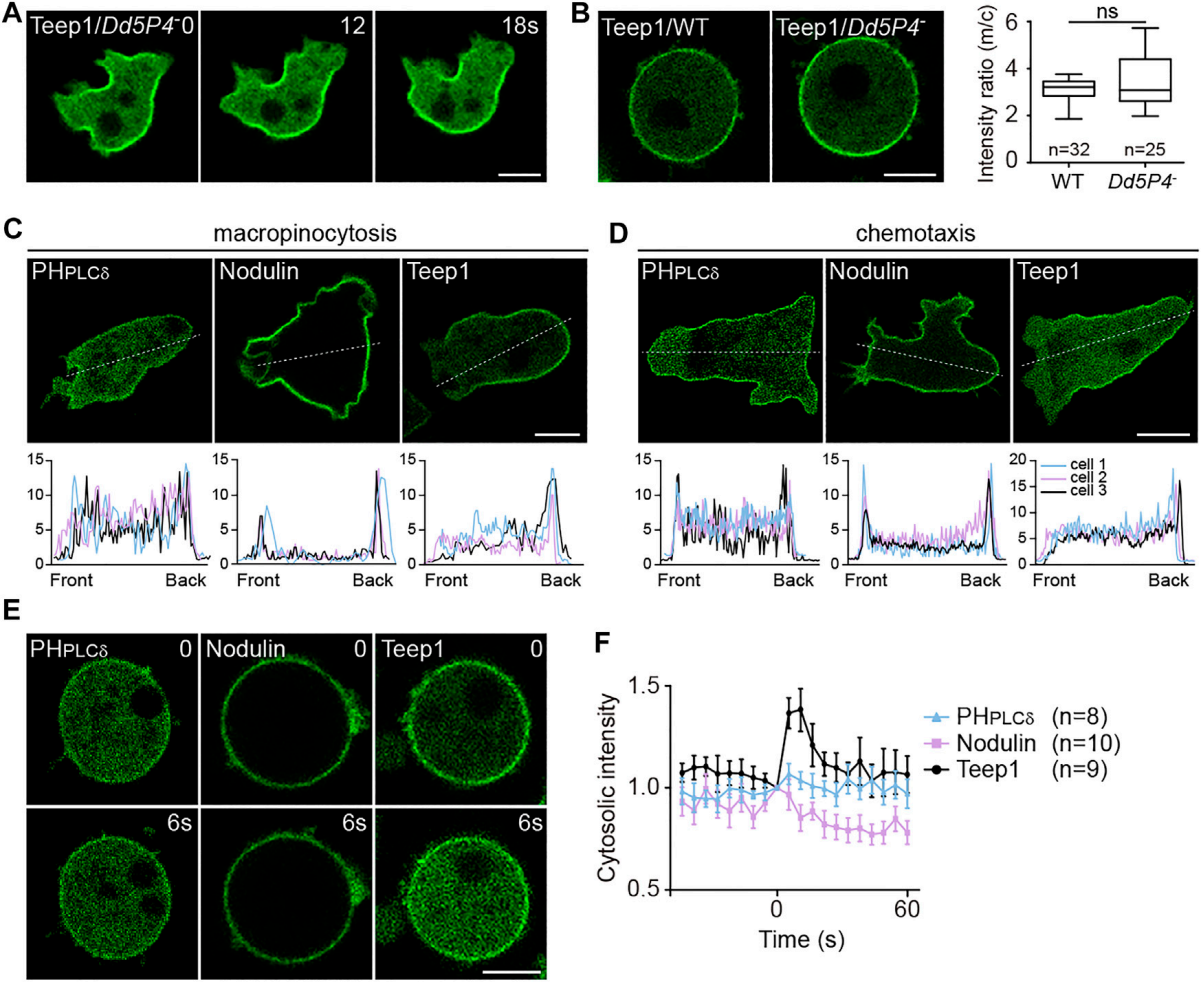
Gradient of PI(3,4)P_2_ or PI(4,5)P_2_ is not sufficient for targeting Teep1. **(A)** Localization of Teep1-GFP in *Dd5P4* ^*−*^ cells. **(B)** Left: Localization of Teep1-GFP in WT and *Dd5P4* ^*−*^ cells treated with 5 μM LatA. Right: Box plot of the membrane-to-cytosol fluorescent intensity ratio of Teep1-GFP in WT and *Dd5P4* ^*−*^ cells. Data was collected from at least two independent experiments. **(C,D)** Top: Distribution of GFP-PH_PLCδ_, GFP-Nodulin, and Teep1-GFP in vegetative cells undergoing macropinocytosis **(C)** or chemotaxis along folic acid gradients **(D)**. Bottom: The corresponding fluorescent intensities along a line connecting front to rear in three representative cells. **(E)** Translocation of GFP-PH_PLCδ_, GFP-Nodulin, and Teep1-GFP in response to folic acid stimulation in the presence of 5 μM LatA (500 μM folic acid was added at time 0). **(F)** Quantification of translocation in response to folic acid stimulation (mean ± SEM). Data was collected from three independent experiments. Scale bar, 5 μm.

We investigated whether gradient of PI(3,4)P_2_ or PI(4,5)P_2_, which has been implicated in back protein localization (Iijima et al., 2004; Gerisch et al., 2011; Li et al., 2018; Yoshioka et al., 2020), underpins the asymmetric distribution of Teep1. First, we examined the involvement of PI(3,4)P_2_. Lack of the inositol 5-phosphatase *Dd5P4*, an OCRL homolog in *Dictyostelium*, was shown to decrease the level of PI(3,4)P_2_ and reduce the membrane-to-cytosol ratio of CynA (Li et al., 2018). We generated *Dd5P4* knockout cells (Supplementary Figure S4A). The newly generated *Dd5P4* ^*−*^ cells were severely defective in macropinocytosis as the original strain (Loovers et al., 2007), and the defects were fully rescued by expression of GFP-Dd5P4 (Supplementary Figure S4B). As seen in fibroblasts containing OCRL mutation (Nández et al., 2014), we observed abnormal actin organization with the presence of numerous actin comets in the cytoplasm of *Dd5P4* ^*−*^ cells (Supplementary Figure S4C). In addition, consistent with the proposed function in PI(3,4)P_2_ production, we observed reduced localization of the PI(3,4)P_2_ sensor TAPP1 in *Dd5P4* ^*−*^ cells (Supplementary Figure S4C). However, the posterior enrichment and membrane association of Teep1 were not affected (Figures 3A,B), indicating that a PI(3,4)P_2_ gradient is not required to restrict the distribution of Teep1.

Next, we investigated PI(4,5)P_2_. The accumulation of PI(4,5)P_2_ at the back of migrating cells and the cleavage furrow have been observed in *Dictyostelium* and other systems (Wong et al., 2005; Janetopoulos and Devreotes, 2006; Keizer-Gunnink et al., 2007; Lokuta et al., 2007; Gerisch et al., 2011; Miao et al., 2019). Consistent with a role in determining the back states of cells, PI(4,5)P_2_ depletion was shown to trigger hyperactivation of cellular protrusions (Miao et al., 2017). However, by comparing Teep1 to two different PI(4,5)P_2_ reporters, PH_PLCδ_ (the PH domain of PLCD1) and Nodulin (the Nlj6-like nodulin domain of AtSfh1) (Ghosh et al., 2015), we noticed that the back-to-front gradient in the plasma membrane association of these reporters was shallower than that of Teep1 (Figures 3C,D). A fraction of PH_PLCδ_ and Nodulin was detected at macropinocytic cups (Figure 3C) or pseudopods of cells migrating under agarose along folic acid gradients (Figure 3D), but Teep1 seemed to be completely excluded from these regions. Furthermore, unlike Teep1, PH_PLCδ_ and Nodulin exhibited minimal chemoattractant-induced translocation (Figures 3E,F). Therefore, even if the PI(4,5)P_2_ gradient is required, it is not sufficient for targeting Teep1. Additional regulatory factors likely exist and may cooperate with changes in PI(4,5)P_2_ to guide the dissociation of Teep1 from protrusions and following stimulation.

### PI(3,5)P_2_ and PI(4,5)P_2_ Jointly Regulate the Localization of Teep1

**FIGURE 4:**
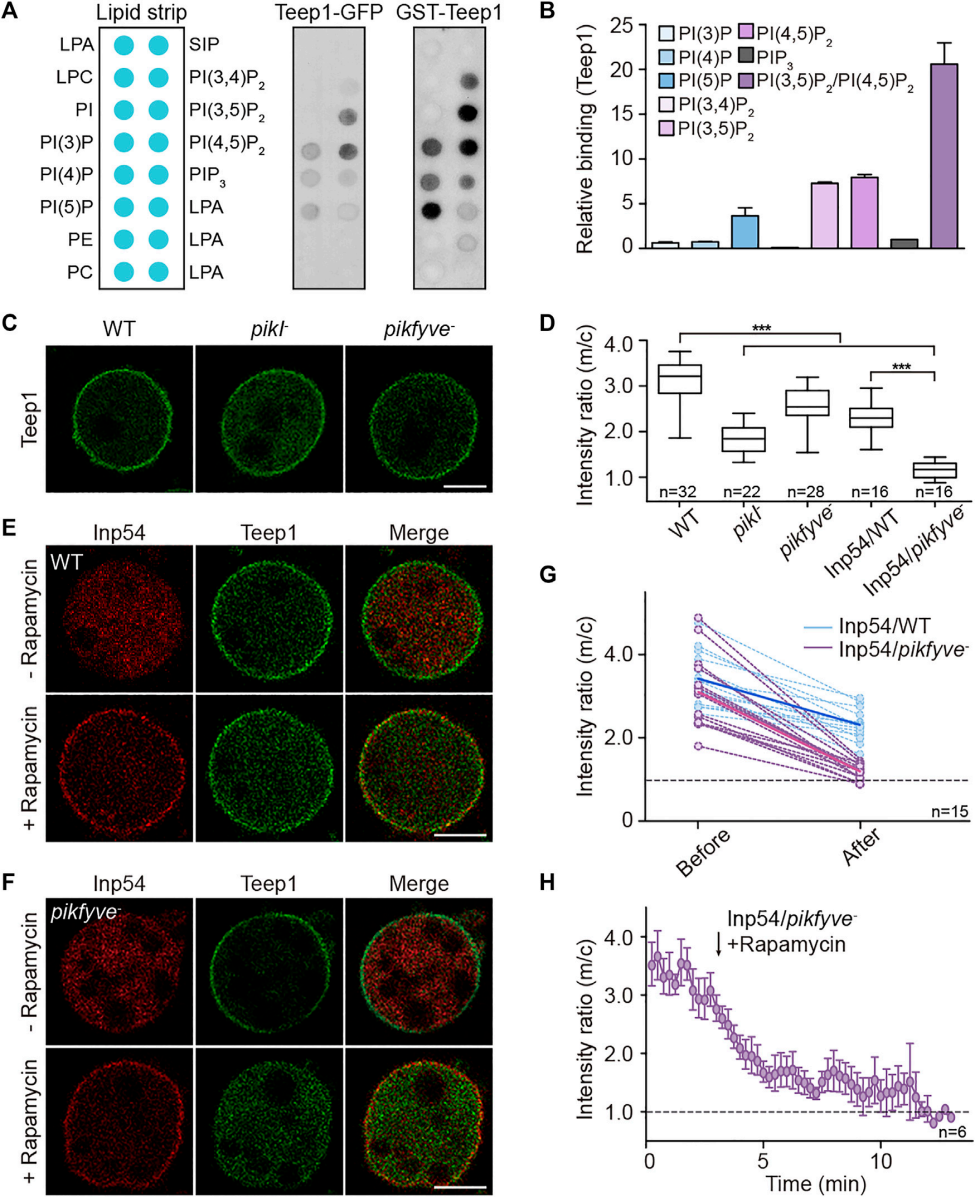
PI(4,5)P_2_ and PI(3,5)P_2_ jointly regulate the localization of Teep1. **(A)** Lipid dot blot assay using Teep1-GFP cell lysate or purified N-terminal fragment of Teep1 (GST-Teep1^N380^). **(B)** Quantification of PIP binding of Teep1 by liposome flotation assay (mean ± SD). Data was from two independent experiments. **(C)** Localization of Teep1-GFP in WT, *pikI* ^*−*^ *,* or *pikfyve* ^*−*^ cells treated with LatA. **(D)** Box plot of the membrane-to-cytosol fluorescent intensity ratios of Teep1-GFP expressed in different cell lines. For Inp54/WT and Inp54/*pikfyve* ^*−*^ cells, images were quantified after rapamycin treatment for 10–15 min. Data was collected from at least two independent experiments. **(E,F)** Localization of Teep1-GFP and mCherry-FRB-Inp54p in LatA-treated WT **(E)** and *pikfyve* ^*−*^ **(F)** cells before and after the addition of rapamycin. **(G)** Quantification of the membrane-to-cytosol fluorescent intensity ratios of Teep1-GFP before and after Inp54 recruitment in WT or *pikfyve* ^*−*^ cells. The dashed lines represent intensity changes in individual cells and the solid lines represent averaged intensity changes. **(H)** Quantification of the membrane-to-cytosol fluorescent intensity ratios of Teep1-GFP before and after rapamycin treatment in *pikfyve* ^*−*^ cells over time (mean ± SEM). Data was from three independent experiments. Scale bar, 5 μm.

We performed dot blot assay to investigate the role of membrane lipids in regulating Teep1 localization. Lipid strips were incubated with cell lysates containing Teep1-GFP. Among 30 different lipids, Teep1 was found to bind only a handful of phospholipids, with a preference for PI(4,5)P_2_ and PI(3,5)P_2_ (Figure 4A; Supplementary Figures S5A,B). PHcrac-GFP, which was included as a control, bound specifically to PI(3,4)P_2_ and PIP_3_ as expected (Supplementary Figure S5C) (Dormann et al., 2004). We purified the N-terminal fragment of Teep1 (Teep1^N380^) containing the PH domains as a GST-fusion protein (Supplementary Figure S5D). When applied to lipid strips, it exhibited a similar binding profile as Teep1-GFP from cell lysates, including weak interactions with several negatively charged phospholipids and a slight preference for PI(4,5)P_2_, PI(3,5)P_2_, and PI5P (Figure 4B). Using the purified fragment, we further assessed the lipid binding selectivity by liposome flotation assays. Although only a small percentage of Teep1 associated with liposomes, increased interaction was observed with those containing PI(4,5)P_2_ or PI(3,5)P_2_, and a modest increase was observed with those containing PI5P (Figure 4B; Supplementary Figure S5E). Including both PI(4,5)P_2_ and PI(3,5)P_2_ in the liposomes further increased the association (Figure 4B; Supplementary Figure S5E). Triple alanine mutations (K11A, K158A, and R174A), which greatly decreased membrane association of Teep1 in cells (Figure 2C), also significantly reduced the binding of purified Teep1 to liposomes containing PI(4,5)P_2_ or PI(3,5)P_2_ (Supplementary Figures S5F,G). Moreover, we found that purified Pten preferentially bound to liposomes containing PI(4,5)P_2_ and PI(3,5)P_2_ (Supplementary Figures S5H–J), suggesting that selectivity for these two lipid species may be a general property of certain back proteins.

The ability of Teep1 and Pten to bind PI(4,5)P_2_ is consistent with the existence of a back-to-front gradient of PI(4,5)P_2_ on the plasma membrane, but binding to PI(3,5)P_2_ is rather unexpected. Although PI(3,5)P_2_ on the plasma membrane has been linked to activities such as mTORC1 activation (Bridges et al., 2012), this lipid is thought to localize primarily to the endolysosomal membrane. Furthermore, PI(3,5)P_2_ is estimated to comprise only a small percentage of total cellular PI (Hasegawa et al., 2017). The liposome flotation experiments described above have a risk of using a lipid concentration that is not physiologically relevant. Therefore, we turned to cell experiments to further test whether gradients of PI(4,5)P_2_ and PI(3,5)P_2_ are required to target Teep1.

We examined the membrane association of Teep1 in cells lacking the kinases responsible for the production of these lipid species. In *Dictyostelium*, as in various other systems, most cellular pools of PI(3,5)P_2_ depend on the activity of the phosphoinositide 5-kinase PIKfyve, which produces PI(3,5)P_2_ by phosphorylating PI3P (Buckley et al., 2019). We generated *pikfyve* ^*−*^ cells (Supplementary Figure S4D). As reported previously, the mutant cells accumulated enlarged endosomes, especially when being shifted to low osmolarity buffer (Buckley et al., 2019), and such defects could be rescued by expression of PIKfyve-GFP (Supplementary Figures S4E,F). In *Dictyostelium*, PI(4,5)P_2_ is produced mainly by the PIP5 kinase PikI (Fets et al., 2014). We expressed Teep1 in WT, *pikfyve* ^*−*^, or *pikI* ^*−*^ cells, treated the cells with LatA, and quantified the membrane-to-cytosol ratio of Teep1. Deleting either kinase partially impaired the plasma membrane association of Teep1 (Figure 4C). The membrane-to-cytosol ratio decreased from 3.1 ± 0.5 in WT to 1.9 ± 0.3 and 2.6 ± 0.4 in *pikI* ^*−*^ and *pikfyve* ^*−*^ cells, respectively (Figure 4D).

To reduce the levels of PI(4,5)P_2_ and PI(3,5)P_2_ simultaneously, we used a chemically inducible dimerization system (Miao et al., 2017). In the system, myristoylated FKBP and a PI(4,5)P_2_-specific phosphatase, Inp54, fused to mCherry-FRB are co-expressed in cells. Upon the addition of rapamycin, Inp54p is rapidly recruited to the plasma membrane, resulting in a significant reduction in PI(4,5)P_2_ on the plasma membrane within 10–15 min (Miao et al., 2017). We transformed the system into WT or *pikfyve* ^*−*^ cells. Teep1 partially disassociated from the plasma membrane when Inp54 was recruited in WT cells (Figure 4E; Supplementary Video S5), with the membrane-to-cytosol ratio reduced to 2.3 ± 0.4 (Figure 4D). In contrast, Inp54 recruitment in *pikfyve* ^*−*^ cells nearly abolished the membrane association of Teep1 (Figure 4F; Supplementary Video S6), with the membrane-to-cytosol ratio reduced to 1.2 ± 0.2 over a time course of approximately 10–12 min (Figure 4H). The magnitude of the response in individual WT and *pikfyve* ^*−*^ cells varied, but the trend was similar (Figure 4G), validating that simultaneous elimination of PI(4,5)P_2_ and PI(3,5)P_2_ blocks the membrane association of Teep1.

Using a newly isolated PI(3,5)P_2_-sensor (Jason King, personal communication), we further analyzed the involvement of PI(3,5)P_2_ in back protein localization. The PI(3,5)P_2_-binding phox homology (PX) domain localized to endosomal structures when expressed as a tandem dimer in cells (Supplementary Figure S5K). Interestingly, when fused with Nodulin, the PX dimer-Nodulin chimeric protein was absent from the macropinocytic cups and distributed to the trailing edge (Supplementary Figure S5K). Collectively, these experiments indicate that PI(4,5)P_2_ and PI(3,5)P_2_ regulate the posterior accumulation of proteins, such as Teep1, by jointly shaping the back state of the plasma membrane.

### A Myotubularin Protein Contributes to Establishing the Potential Back-to-Front Gradient of PI(3,5)P_2_

The above results suggest a potential back-to-front gradient of PI(3,5)P_2_ that acts together with PI(4,5)P_2_ gradient to regulate back events. The gradient of PI(4,5)P_2_ on the plasma membrane is thought to be established by PI5K- and Pten-mediated production at the back, as well as PI3K- and PLC-mediated removal from the front (Funamoto et al., 2002; Iijima and Devreotes, 2002; Keizer-Gunnink et al., 2007; Miao et al., 2019). We speculated that kinases or phosphatases responsible for producing or degrading PI(3,5)P_2_ may also be distributed in a polar matter. We found that PIKfyve-GFP (Buckley et al., 2019) was localized in the cytoplasm and on Rab7A-positive compartments (Supplementary Figure S4F), suggesting that PI(3,5)P_2_ production may not be spatially restricted. We then examined enzymes responsible for PI(3,5)P_2_ turnover. This process is proposed to be catalyzed by the Sac1-related phosphatase Fig4 or myotubularin family of phosphatases (Schaletzky et al., 2003; Tronchère et al., 2004; Vaccari et al., 2011; Hasegawa et al., 2017). *Dictyostelium* genome encodes one Fig4 protein (DDB_G0281427) and nine putative myotubularins, which we named Mtm1-9 (Supplementary Figure S6A). We tagged each of these proteins with GFP and examined their localization. Intriguingly, one of the myotubularin phosphatases, Mtm6, exhibited polarized distribution.

**FIGURE 5:**
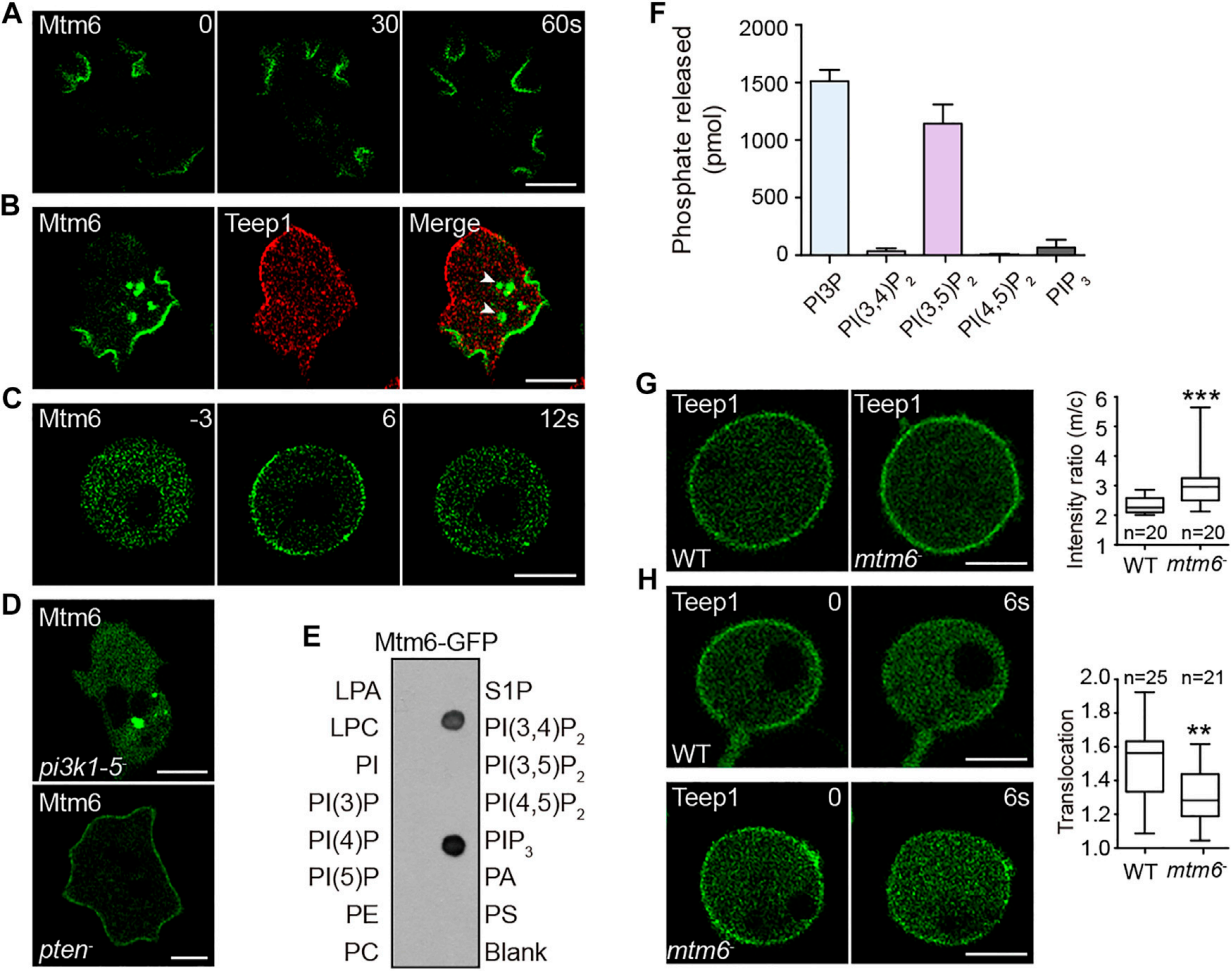
Mtm6 contributes to the establishment of a potential back-to-front gradient of PI(3,5)P_2_. **(A)** Localization of Mtm6-GFP in WT cells during macropinocytosis. **(B)** Localization of Mtm6-GFP and Teep1-RFP in WT cells. Arrow heads point to newly formed macropinosomes. **(C)** Mtm6-GFP translocation in response to cAMP stimulation in LatA-treated WT cells (1 μM cAMP was added at time 0). **(D)** Localization of Mtm6-GFP in *pi3k1-5* and *pten* null cells. **(E)** Lipid dot blot assay using cell lysates expressing Mtm6-GFP. **(F)** *In vitro* phosphatase assay using Mtm6-GFP immunoprecipitated from cell lysates (mean ± SD). Data was collected from three independent experiments. **(G)** Localization of Teep1-GFP in differentiated WT and *mtm6* ^*−*^ cells treated with LatA. Box plot shows the membrane-to-cytosol fluorescent intensity ratios of Teep1-GFP. Data was from at least two independent experiments. **(H)** Translocation of Teep1-GFP in LatA-treated WT or *mtm6* ^*−*^ cells in response to cAMP stimulation (1 μM cAMP was added at time 0). Box plot shows the translocation efficiency of Teep1-GFP at 6 s after the addition of cAMP. Data was collected from at least three independent experiments. Scale bar, 5 μm.

Mtm6-GFP localized selectively at the leading edge of migrating cells, as well as macropinocytic cups (Figure 5A; Supplementary Video S7). When co-expressed with Teep1, Mtm6-GFP and Teep1-RFP exhibited mutually exclusive distribution on the plasma membrane (Figure 5B). As with other leading-edge proteins, Mtm6 responded to chemoattractant stimulation (Supplementary Figure S6B), and the translocation could occur in the presence of LatA (Figure 5C). The localization pattern of Mtm6 prompted us to examine whether this relies on interaction with the classic leading-edge signal, PIP_3_. We found that the plasma membrane localization of Mtm6 was abolished in a quintuple PI3K mutant (Hoeller et al., 2013) but greatly enhanced in *pten* ^*−*^ cells (Iijima and Devreotes, 2002) (Figure 5D). Furthermore, when incubated with phosphatidylinositol phosphate (PIP) strips, Mtm6-GFP bound specifically to PIP_3_ and PI(3,4)P_2_, with a preference for PIP_3_ (Figure 5E). Considering that PI(3,4)P_2_-sensing proteins only weakly label the macropinocytic cups and do not change localization in response to stimulation (Yang et al., 2021), PIP_3_ is likely primarily responsible for recruiting Mtm6 to the plasma membrane at the leading edge. PIP_3_ is converted into PI(3,4)P_2_ during macropinosome formation (Yang et al., 2021). The ability of Mtm6 to interact with PI(3,4)P_2_ may account for its additional distribution on nascent macropinosomes (Figure 5B).

We reasoned that leading edge-localized Mtm6 may mediate PI(3,5)P_2_ removal from the front and facilitate the establishment of a back-to-front gradient of PI(3,5)P_2_. In line with this model, a malachite-based assay revealed that Mtm6-GFP immunoprecipitated from cell lysates exhibited high activity against PI(3,5)P_2_ (Figure 5F). Mtm6-GFP also was able to degrade PI3P (Figure 5F), and the significance of this activity is unclear. We then examined whether Mtm6 is involved in regulating the localization of Teep1. To this end, we generated *mtm6* knockout cells (Supplementary Figures S6C–E). Opposite to the effect of deleting *pikfyve* (Figures 4C,D), deleting *mtm6* resulted in a modest increase in the membrane targeting efficiency of Teep1 (Figure 5G). The chemoattractant-induced membrane-to-cytoplasm translocation was also partially affected by *mtm6* deletion (Figure 5H). Taken together, these experiments reveal a possible mechanism for establishing a reverse PI(3,5)P_2_ gradient on the plasma membrane, which in turn regulates the localization of back proteins.

### Teep1 Deletion Impairs Cell Motility

To analyze the function of Teep1, we generated *teep1* knockout cells (Supplementary Figures S7A,B). When initially assessed on bacterial lawns, we found that plaque growth of *teep1* ^*−*^ cells was indistinguishable from that of WT, indicating that bacterial uptake, digestion, and multicellular development were not affected by *teep1* deletion (not shown). Consistently, when plated on non-nutrient agar, *teep1* ^*−*^ cells differentiated and formed streams of migrating cells and fruiting bodies similar to WT (Supplementary Figure S7C). Cell proliferation in liquid medium was similarly unaffected by *teep1* deletion. Therefore, *teep1* deletion does not grossly affect cell growth or development.

**FIGURE 6:**
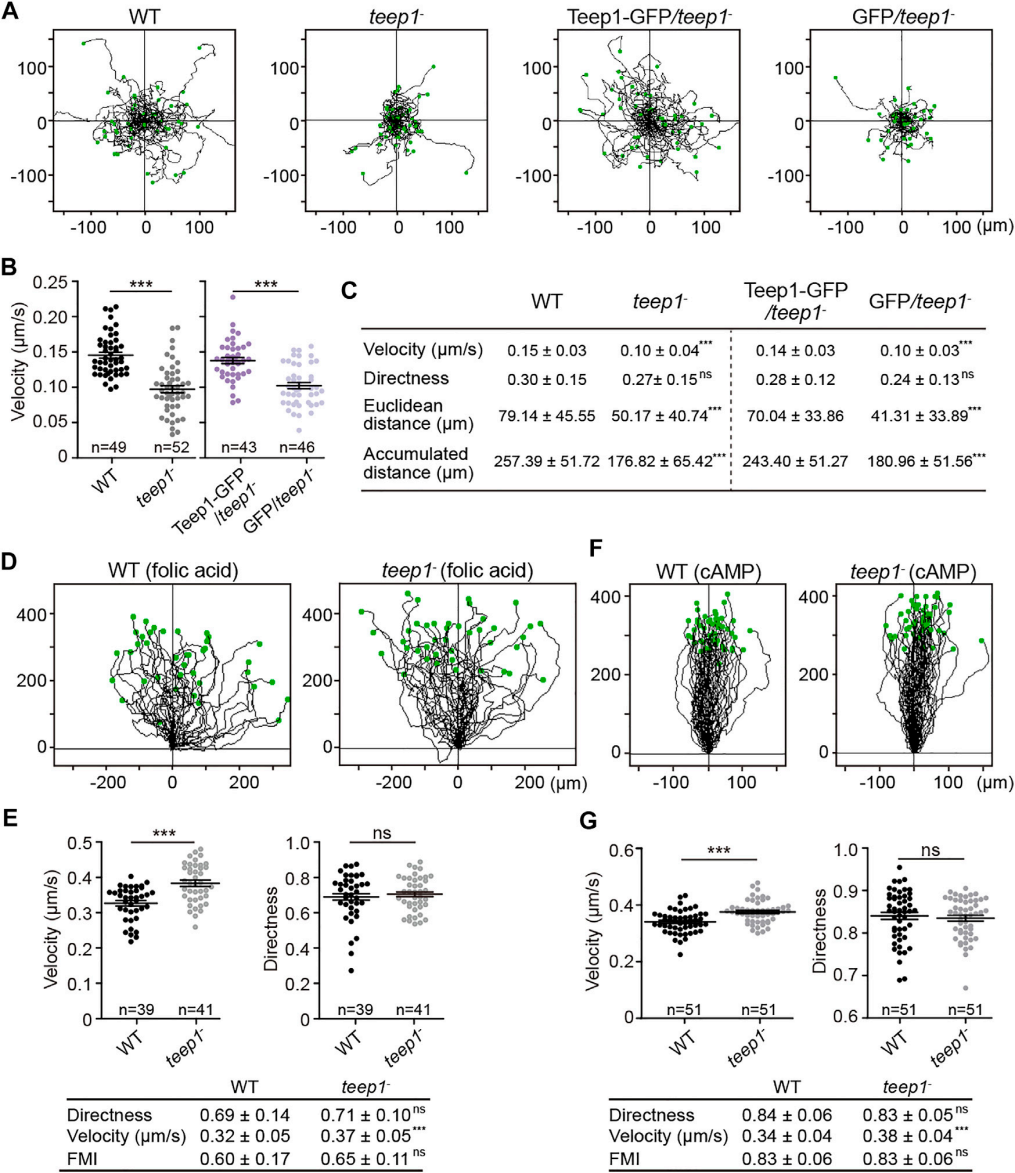
Deletion of *teep1* impairs cell motility. **(A)** Trajectories of randomly migrating vegetative cells. **(B)** Quantification of the speed of random movement. The scatter plots show data points with means and SEM. **(C)** Summary of random motility parameters (mean ± SD). **(D)** Trajectories of WT and *teep1* ^*−*^ cells chemotaxing under agarose along folic acid gradients **(E)** Top: Quantification of velocity and directness of cells chemotaxing along folic acid gradients. Bottom: summary of chemotaxis parameters (mean ± SD). **(F)** Trajectories of WT and *teep1* ^*−*^ cells chemotaxing under agarose along cAMP gradients. **(G)** Top: Quantification of velocity and directness of cells chemotaxing along cAMP gradients. Bottom: summary of chemotaxis parameters (mean ± SD). FMI, forward migration index. Data was collected from three independent experiments.

We examined the kinetics of cell migration quantitatively. In random motility assays, Teep1 deletion led to a significant decrease in the speed of cell movement. Tracks of individual cells demonstrated that WT cells moved much further from starting points compared to *teep1* ^*−*^ cells (Figure 6A; Supplementary Video S8). The average speed of WT cells was 9.0 ± 1.8 μm/min, whereas that of *teep1* ^*−*^ cells was 6.0 ± 2.4 μm/min (Figures 6B,C). Expression of Teep1-GFP, but not GFP, in the null background restored motility to WT level (Figures 6A–C). However, to our surprise, *teep1* ^*−*^ cells did not exhibit an apparent defect in directed migration. When cells were exposed to gradients of folic acid or cAMP (Tweedy et al., 2016), the *teep1* ^*−*^ cells migrated up the gradients with comparable directness and chemotactic index to WT cells and even exhibited a slight increase in the average speed (Figures 6D–G). Therefore, Teep1 appears to be involved in cell motility regulation, but its function can be bypassed by exposing cells to chemoattractant gradients.

We speculated that the decreased random motility observed in *teep1* ^*−*^ cells might result from a dysfunction in myosin II-based contractility, which is a well-known back event. However, the dynamic enrichment of myosin II at retraction sites did not appear to be affected by *teep1* deletion (Supplementary Figure S7D). To further investigate the function of Teep1, we sought out its binding partners. Immunoprecipitation and mass spectrometry analysis revealed that Teep1 interacted with the membrane-cortex linker protein, TalinB (TalB) (Supplementary Figure S7E). We verified this interaction by co-immunoprecipitation and colocalization experiments; Teep1-RFP co-precipitated with GFP-TalB (Supplementary Figure S7F). Furthermore, GFP-TalB was recruited to the cell periphery by Teep1-RFP in LatA-treated cells (Supplementary Figure S7G). A newly identified leading-edge protein, Leep1, which was included as a control, did not interact with TalB (Yang et al., 2021). Cell adhesion assay did not reveal an apparent defect in *teep1* ^*−*^ cells (Supplementary Figure S7H); therefore, whether the interaction with TalB and the function of TalB in substrate adhesion or force transmission (Tsujioka et al., 2004; Tsujioka et al., 2008; Plak et al., 2016) underlie the requirement for Teep1 in random motility needs to be further investigated.

## Discussion

In this study, by screening for PH domain-containing proteins that exhibit polarized distribution, we identified a novel trailing-edge protein, Teep1 (Figure 1). We showed that Teep1 exhibits identical dynamic behavior to the well-known trailing-edge marker protein, Pten, including dissociating selectively from protrusions and macropinocytic cups, distributing uniformly on the plasma membrane in LatA-treated cells, and responding to global chemoattractant stimulation by transiently falling off the membrane. Characterization of the molecular mechanisms that control the localization of Teep1 allowed us to gain fresh insights into how the back state is defined and regulated.

Several lines of evidence indicate that back-to-front gradients of PI(4,5)P_2_ and PI(3,5)P_2_ regulate the posterior accumulation of proteins, such as Teep1, by jointly shaping the back state of the plasma membrane. First, we found that a potential charged surface formed by the two N-terminal PH domains is both necessary and sufficient for targeting Teep1 to the rear of cells (Figure 2; Supplementary Figure S3). Second, Teep1 binds to negatively charged phospholipids *in vitro*, with a preference for PI(4,5)P_2_ and PI(3,5)P_2_ (Figure 4; Supplementary Figure S5). Third, deleting the kinase responsible for producing either PI(4,5)P_2_ or PI(3,5)P_2_ partially impairs the plasma membrane association of Teep1, whereas simultaneous elimination of PI(4,5)P_2_ and PI(3,5)P_2_ nearly blocks its membrane localization (Figure 4). In addition, a chimeric sensor composed of PI(4,5)P_2_- and PI(3,5)P_2_-recognition modules exhibits a posterior enrichment (Supplementary Figure S5). Finally, we showed that a myotubularin phosphatase, Mtm6, which is capable of degrading PI(3,5)P_2_, likely mediates the removal of PI(3,5)P_2_ from the front and the formation of a reverse PI(3,5)P_2_ gradient (Figure 5). Consistently, deleting *mtm6* slightly increases the membrane targeting efficiency of Teep1 (Figure 5). Mtm6 is recruited to the leading-edge via interaction with PIP_3_ (Figure 5). This could represent another example of crosstalk between front and back signals, which has been proposed to ensure their spatial separation (Li et al., 2018; Matsuoka and Ueda, 2018).

Our study raises a few intriguing questions that remain to be answered. First, Teep1 seems to interact with both PI(4,5)P_2_ and PI(3,5)P_2_, suggesting that its localization may be regulated by a coincidence-detection mechanism, but we do not yet understand how this is achieved at the molecular level. We found that PI(4,5)P_2_ or PI(3,5)P_2_ depletion has different impacts on the localization of Teep1 bearing mutations in either of the PH domains (Supplementary Figure S3F). This observation suggests that the two PH domains may have different binding selectivity, providing a means for coincidence detection. Previous studies have revealed that PH domains possess both canonical and non-canonical PIP binding sites, which allow them to associate with more than one PIP molecule (Ceccarelli et al., 2007; Anand et al., 2012; Jian et al., 2015). The PH domains of Teep1 contain both types of binding sites (Supplementary Figure S3C). Thus, coincidence detection may be achieved alternatively by using one PH domain to interact with PI(4,5)P_2_ and PI(3,5)P_2_ simultaneously. Assessing the lipid binding specificity of individual PH domain in cells or *in vitro* assays is needed to distinguish between these possibilities. However, the expression of the two PH domains is poor when expressed separately in cells or as recombinant proteins, precluding further analysis.

Second, whether reverse gradients of PI(4,5)P_2_ and PI(3,5)P_2_ provide a general mechanism for targeting proteins to the trailing edge requires further investigation. Using purified proteins, we found that Pten also binds preferentially to liposomes containing PI(4,5)P_2_ and PI(3,5)P_2_ (Supplementary Figures S5H–J), suggesting that such selectivity may be applicable to other back proteins in addition to Teep1. Intriguingly, simultaneous depletion of PI(4,5)P_2_ and PI(3,5)P_2_ by recruitment of Inp54 in *pikfyve* ^*−*^ cells not only results in Teep1 dissociation but also causes substantially increased blebbing (Supplementary Figure S8; Supplementary Video S9), a phenotype usually associated with increased contractility or defects in membrane-cortex adhesion or cortical integrity (Stossel et al., 2001; Bovellan et al., 2014; Zatulovskiy et al., 2014; Ramalingam et al., 2015; Ruprecht et al., 2015; Srivastava et al., 2020). In contrast, recruitment of Inp54 in WT cells causes mainly fan-like or oscillatory behaviors as reported previously (Miao et al., 2017). Considering that the blebbing phenotype is not observed in *teep1* ^*−*^ cells and that a number of membrane-cortex linkers or proteins involved in cortex assembly exhibit posterior accumulation (Faix et al., 2001; Kee et al., 2012; Tsujioka et al., 2012; Litschko et al., 2019), the removal of PI(4,5)P_2_ and PI(3,5)P_2_ may have a greater impact on the back state of cells by affecting the membrane association of multiple proteins and reducing the threshold for blebbing.

Finally, our study, together with previous findings, indicates that the signaling network that determines the back state of cells likely consists of redundant or parallel pathways. A gradient of PI(4,5)P_2_/PI(3,5)P_2_ is unlikely to be the only back signal. For example, the back-localized CynA protein has been shown to interact selectively with PI(3,4)P_2_ (Li et al., 2018). The localization of some other back proteins, including myosin II and TalA, has been shown to rely on actin cytoskeleton (Levi et al., 2002; Tsujioka et al., 2012). It will be of great interest in future studies to investigate how different pathways integrate in space and time to regulate the back activities of cells.

## Materials and Methods

### Cell Culture, Transformation, and Differentiation

WT cells were derived from the Ax2 (Ka) axenic strain (Bloomfield et al., 2008). All gene deletion cell lines were generated in Ax2. WT and gene deletion cells were cultured in HL5 medium (Formedium HLF3) supplemented with antibiotics. The *pikI* ^***−***^ cells were cultured on bacterial lawns (*Klebsiella aerogenes*) and transferred into HL5 before use in experiments (Fets et al., 2014). Cells carrying expression constructs were maintained in HL5 containing G418 (10–40 μg/ml), Hygromycin (50 μg/ml), or both as needed. Development on non-nutrient agar or with cAMP pulses was performed as described before (Cai et al., 2014).

### Gene Disruption and Plasmid Construction

To make knockout constructs for *teep1*, *mtm6*, *pikfyve*, and *Dd5P4* deletion, a blasticidin S resistance (BSR) cassette was inserted into pBlueScript II SK+ to generate pBlueScript-BSR (Kimmel and Faix, 2006). 5′ and 3′ arms were PCR-amplified from genomic DNA with primers listed in Table 1 and cloned upstream and downstream of the BSR cassette, respectively. The resulting disruption cassette was electroporated into Ax2. Gene disruption was confirmed by resistance to blasticidin (10 μg/ml), PCR, or Southern Blotting.

**TABLE 1:** Plasmids and primers used in this study. Each primer is designated as forward (F) or reverse (R).

Usage	Plasmid backbone	Sequence, 5′-3′
Expression in *Dictyostelium* cells		
Teep1-GFP	pDM323	F: CCG​GAG​CTC​ATG​ATA​TCA​ATC​GAA​GAA​AAT​ATT​AAA​TAC
		R: CTA​GCT​AGC​AAA​TAA​TTT​TAC​AGA​ACA​AGT​GCC​GCA​ATA​AG
Teep1-ΔLIM-GFP	pDM323	F: GCT​CTA​GAA​TGG​GTG​GAA​TCG​ACG​AAG​ATG​G
		R: ATA​AGA​ATG​CGG​CCG​CTT​TTG​CAT​TTG​ATT​TAT​TTG​AAT​TTA​TTG
Teep1-PH-GFP	pDM323	F: CCG​GAG​CTC​ATG​ATA​TCA​ATC​GAA​GAA​AAT​ATT​AAA​TAC
		R: CTA​GCT​AGC​AGA​TTT​TGT​AAG​GCT​ACG​ATT​GTT​ATC
Teep1-LIM-GFP	pDM317	F: CCG​GAG​CTC​CCA​ACT​TCA​ACA​CCA​GTT​AAA​TCA​ACA​TCA​C
		R: CTA​GCT​AGC​AAA​TAA​TTT​TAC​AGA​ACA​AGT​GCC​GCA​ATA​AG
Teep1^N411^-GFP	pDM323 or pCV5	F: CCG​GAG​CTC​ATG​ATA​TCA​ATC​GAA​GAA​AAT​ATT​AAA​TAC
		R: CTA​GCT​AGC​CTC​TTT​TGT​ATT​GGT​TGT​TGT​TGT​AG
GFP-Teep1-ΔPH	pDM317	F: CCG​GAG​CTC​TTA​GCA​ACT​CCA​AAT​GAA​ATC​ACA​AGA​C
		R: CTA​GCT​AGC​AAA​TAA​TTT​TAC​AGA​ACA​AGT​GCC​GCA​ATA​AG
Teep1N411^K11A^-GFP	pDM323	F: CCGGAGCTC ATG​ATA​TCA​ATC​GAA​GAA​AAT​ATT​AAA​TAC​GCA
		R: CTA​GCT​AGC​CTC​TTT​TGT​ATT​GGT​TGT​TGT​TGT​AG
Teep1N411^R28A^-GFP	pDM323	F: CAT​TGT​GTA​TTT​AAA​AAT​AG
		R: TGC​CTT​TTT​CCA​TGA​TTT​ACC​ATC​AGA​TG
Teep1N411^K158A^-GFP	pDM323	F: TAT​ACA​TCA​TCA​GGT​ACA​TTT​AGA​AAA​AC
		R: TGC​TTT​TAA​CCA​ACC​TTT​ATG​ATC​TGA​TG
Teep1N411^R174A^-GFP	pDM323	F: TGG​TTC​GTA​CTA​AAG​GAT​TTA​GTA​CTC
		R: TGC​CTT​TTT​CCA​TTG​AAG​TGT​TTT​TC
Teep1N411^K11E^-GFP	pDM323	F: CCG​GAG​CTC​ATG​ATA​TCA​ATC​GAA​GAA​AAT​ATT​AAA​TAC GAA GAAG
		R: CTA​GCT​AGC​CTC​TTT​TGT​ATT​GGT​TGT​TGT​TGT​AG
Teep1N411^R28D^-GFP	pDM323	F: CAT​TGT​GTA​TTT​AAA​AAT​AG
		R: ATC​CTT​TTT​CCA​TGA​TTT​ACC​ATC​AGA​TG
Teep1N411^R174D^-GFP	pDM323	F: TGG​TTC​GTA​CTA​AAG​GAT​TTA​GTA​CTC
		R: ATC​TGC​CTT​TTT​CCA​TTG​AAG​TGT​TTT​TC
Teep1N411^K11AR28A^-GFP	pDM323	As above
Teep1N411^K11AK158A^-GFP	pDM323	As above
Teep1N411^K11AR174A^-GFP	pDM323	As above
Teep1N411^R28AR174A^-GFP	pDM323	As above
Teep1N411^K158AR174A^-GFP	pDM323	As above
Teep1N411^K11AR28AK158A^-GFP	pDM323	As above
Teep1N411^K11AR28AR174A^-GFP	pDM323	As above
Teep1N411^K11AK158AR174A^-GFP	pDM323	As above
Teep1-RFP	pDM451	F: CCG​GAG​CTC​ATG​ATA​TCA​ATC​GAA​GAA​AAT​ATT​AAA​TAC
		R: CTA​GCT​AGC​AAA​TAA​TTT​TAC​AGA​ACA​AGT​GCC​GCA​ATA​AG
Mtm6-GFP	pDM323	F: CCG​GAG​CTC​ATG​AAT​CAA​CAA​CAG​ATT​GTT​AAT​GAT​C
		R: CTA​GCT​AGC​AAT​ATC​TTT​TAA​ATC​ATT​AAT​AAT​TGA​AG
PhdB-GFP	pDM323	F: GGA​GCT​CAT​GCA​TAC​AGG​AGA​ATA​C
		R: CGG​ACT​AGT​TAA​AAA​TTG​AGA​AAT​ATA​ATA​AT
TAPP1-GFP	pDM323	F: CCG​GAG​CTC​ATG​CCT​TAT​GTG​GAT​CGT​CAG
		R: CTA​GCT​AGC​CAC​GTC​ACT​GAC​CGG​AAG​GC
GFP-Dd5P4	pDM317	F: CCG​GAG​CTC​ATG​GGT​GAT​ATT​CAA​AAT​ACA​GAT​AAT​ATA​G
		R: CTA​GCT​AGC​ATT​AAT​TAA​ATC​TTT​TGA​AAT​TAA​AAA​ATG
Pikfyve-GFP	pDM323	F: CCG​GAG​CTC​ATG​GCA​GAA​TCA​TTC​CAA​CAA​TTA​GG
		R: CTA​GCT​AGC​TTT​ATT​AAT​TTG​TTG​GAC​TTG​TCT​TTG​ATT​TAT​ATT​TCC
PX-PX-GFP	pDM323	F1: CCG​GAG​CTC​ATG​AAT​AGA​AAT​AAT​GAA​ATT​TAT​ATC
		R1: CTA​GCT​AGC​TGA​ACC​TGA​ACC​TGA​ACC​TGA​ACC​GTT​TTG​ACC​TTC​GTC​TCT​TTT​AAG
		F2: CTA​GCT​AGC​AAT​AGA​AAT​AAT​GAA​ATT​TAT​ATC
		R2: CGG​ACT​AGT​GTT​TTG​ACC​TTC​GTC​TCT​TTT​AAG
GFP-PX-PX-Nodulin	pDM323	F1: CCG​GAG​CTC​ATG​AAT​AGA​AAT​AAT​GAA​ATT​TAT​ATC
		R1: CTA​GCT​AGC​TGA​ACC​TGA​ACC​TGA​ACC​TGA​ACC​GTT​TTG​ACC​TTC​GTC​TCT​TTT​AAG
		F2: CTA​GCT​AGC​AAT​AGA​AAT​AAT​GAA​ATT​TAT​ATC
		R2: CGG​ACT​AGT​GTT​TTG​ACC​TTC​GTC​TCT​TTT​AAG
		F3: CGG​ACT​AGT​GTT​AGG​CTA​TCA​AAA​GAC​GTT​CCA​CGC
		R3: CGG​ACT​AGT​GAA​TCC​GAA​AAA​CAG​CTT​C
RFP-Rab7A	pDM449	F: CGG​GAG​CTC​ATG​GCC​ACA​AAG​AAA​AAG​G
		R: CGG​ACT​AGT​ACA​ACA​ACC​TGA​TTT​AGC​TGG
GFP-Nodulin	pDM317	F: TGC​TCT​AGA​GTT​AGG​CTA​TCA​AAA​GAC​GTT​CCA​CGC
		R: CAC​GGT​ACC​GAA​TCC​GAA​AAA​CAG​CTT​C
GFP-Myosin II	pCV5	F: CCG​GAG​CTC​AAT​CCA​ATT​CAT​GAT​AGA​ACT​TCA​GAT​TAT​C
		R: CAG​GCT​CGA​GTT​AAG​CTT​TGA​AAC​CAC​CAA​AGA​AAT​CGG​C
Generation of knockout cell		
*teep1* knockout	pBluescript-BSR	Insert 1 F: CAC​GGT​ACC​GAT​ACC​ATC​ATC​GAT​GAT​ATC​AC
		Insert 1 R: GAG​AAG​CTT​CCA​TCA​GAT​GAC​AAA​ACT​GAA​AG
		Insert 2 F: CGG​ACT​AGT​GTA​AAG​TTA​CAA​CAC​CAA​TTT​CTA​CAC
		Insert 2 R: CGG​CGG​CCG​CGT​TAA​CAG​CTT​GGA​AAT​CAT​CCA​TTG
*Dd5P4* knockout	pBluescript-BSR	Insert 1 F: CAC​GGT​ACC​GAT​TAA​ACA​AAA​TGA​AAC​GCA​ACT​TTT​C
		Insert 1 R: GAG​AAG​CTT​CTG​TAT​TTT​GAA​TAT​CAC​CCA​TTT​TG
		Insert 2 F: CGC​GGA​TCC​GAT​GCT​ACA​ACT​GTT​AAA​AAG​AAA​GCT​G
		Insert 2 R: CCG​GCG​GCC​GCG​TTG​TAA​AAA​AGA​CAT​TAA​TTG​GTT​TCT​C
*pikfyve* knockout	pBluescript-BSR	Insert 1 F: CAC​GGT​ACC​CAA​AAC​TAA​ATA​TCT​TTT​TGA​TAC​GTG
		Insert 1 R: GAG​GTC​GAC​CTG​CCA​TTA​TTA​GGA​TTA​TTT​GAA​C
		Insert 2 F: CGG​ACT​AGT​CAA​GTC​CAA​CAA​ATT​AAT​AAA​TAA​C
		Insert 2 R: CGG​CGG​CCG​CCA​TTT​GAT​ATG​TTT​AAA​TCA​GAT​AAT​GG
*mtm6* knockout	pBluescript-BSR	Insert 1 F: GAC​GTC​GAC​GAA​TAA​TAT​AGC​CAG​AGT​TTT​TTA​TTG​AAT​AG
		Insert 1 R: GAG​AAG​CTT​CAA​TGC​AAC​CAT​ATT​ATC​ATT​CAT​TGC
		Insert 2 F: CGG​ACT​AGT​GAA​GAA​GAG​AGA​TCT​CCA​ATT​TTT​CAA​C
		Insert 2 R: CGG​CGG​CCG​CAA​TAT​CTT​TTA​AAT​CAT​TAA​TAA​TTG​AAG​TAG​G
Expression in bacteria		
GST-Teep1^N380^	pGEX-6P-1	F: CGC​GGA​TCC​ATG​ATA​TCA​ATC​GAA​GAA​AAT​ATT​AAA​TAC
		R: CTA​GCT​AGC​TGT​ATT​TAT​TGA​TGG​TGT​TGA​AG
GST-Teep1^N380-K11AK158AR174A^	pGEX-6P-1	F: CGC​GGA​TCC​ATG​ATA​TCA​ATC​GAA​GAA​AAT​ATT​AAA​TAC
		R: CTA​GCT​AGC​TGT​ATT​TAT​TGA​TGG​TGT​TGA​AG
GST-Pten	pGEX-6P-1	F: CGG​AGA​TCT​ATG​AGT​AAT​TTA​TTA​AGA​GTT​GCA​GTC​TC
		R: GAC​CCT​CGA​GAC​TTG​AGC​TAT​TTG​AAG​AAG​TTT​CAC​TG
Other plasmids for expression in *Dictyostelium* cells		
mCherry-FRB-Inp54	Peter Devreotes Laboratory, Johns Hopkins University	
PKBR1^N150^-FKBP	Peter Devreotes Laboratory, Johns Hopkins University	
PHcrac-GFP	Peter Devreotes Laboratory, Johns Hopkins University	
mKikGR-tPH_CynA_	Peter Devreotes Laboratory, Johns Hopkins University	
LimEΔcoil-GFP	Peter Devreotes Laboratory, Johns Hopkins University	
GFP-PH_PLCδ_	Miho Iijima Laboratory, Johns Hopkins University	
Pten-GFP	Miho Iijima Laboratory, Johns Hopkins University	
RFP-Myosin II	Douglas Robinson Laboratory, Johns Hopkins University	
LimEΔcoil-RFP	Douglas Robinson Laboratory, Johns Hopkins University	

To generate constructs expressing GFP- or RFP-fusion proteins, DNA fragments were PCR-amplified using primers listed in Table 1 and cloned into pDM vectors (Veltman et al., 2009) containing a multiple cloning site. For expression of GST-fusion proteins in bacteria, DNA fragments were cloned into pGEX-6P-1 vector at BamHI and XhoI sites. To express PX-PX dimer, the first PX domain was amplified from *Dictyostelium* cDNA using primers F1 and R1 and inserted into pDM323 at SacI and NheI sites; the second PX domain was amplified using primers F2 and R2 and inserted at NheI and SpeI sites. A flexible linker (GSGSGSGS) was added between the two PX domains. To express the chimeric sensor, the Nodulin domain of Atsfh1 was amplified using primers F3 and R3 and inserted into the PX dimer-GFP construct at the SpeI site.

### Imaging

To image the localization of fluorescent proteins, 10^5^ cells were plated in 8-well coverslip chambers (Lab-Tek; NalgenNunc) filled with HL5 or LoFlo medium (Formedium) and allowed to adhere. Images were acquired on a Zeiss 880 inverted microscope equipped with a 40 ×/.95 or 63 ×/1.4 oil-immersion objective. For LatA treatment, cells were incubated with 5 μM LatA (Enzo Life Sciences BML-T119-0100) for 5–10 min before imaging. To image the localization of Teep1 in LatA-treated cells in response to cAMP gradient, a μ-Slide Chemotaxis chamber (ibidi) was utilized. Cell loading was conducted following the manufacturer’s instruction. Before imaging, 1 μl of LatA (50 μM) was added via port A. To image protein translocation in response to cAMP stimulation, differentiated cells were stimulated with 1 μM cAMP. To image protein translocation in response to folic acid, vegetative cells were incubated in development buffer (DB) for 30 min before the addition of 200–500 μM folic acid. The Inp54 recruitment experiment was performed as described previously (Miao et al., 2017). In brief, growth-stage cells were placed in coverslip chamber and allowed to adhere for 10–15 min. After cells adhered, the medium was replaced with 450 μl DB, and 50 μl rapamycin solution was added to a final concentration of 5–10 μM.

Image analyses were performed using ImageJ. The membrane-to-cytosol fluorescent intensity ratio was determined by dividing the total fluorescence intensity at the cell periphery by that in the cytosol as described previously (Nguyen et al., 2014). Translocation dynamics was quantified by measuring the changes of cytosolic fluorescent intensity over time (the intensity at the last time point before the addition of stimuli was normalized to 1). Translocation efficiency in Figure 5H was determined by dividing the cytosolic fluorescent intensity at the peak of translocation by that at the last time point before stimulation.

### Migration Assays

For random motility assay, vegetative cells were seeded in culture plate in HL5 and allowed to adhere for 4 h. Before imaging, the medium was replaced with fresh HL5. Images were acquired at 20 s intervals with phase illumination using a 10×/.45 or 20×/.8 objective. Under-agarose folic acid chemotaxis assay was performed as described before (Woznica and Knecht, 2006). Briefly, after setting of the agarose containing 10 μM folic acid, one trough of 5 mm wide was cut and filled with vegetative cells. Cells were allowed to migrate for 4–7 h. Images were acquired at 20 s intervals using a 10 ×/.45 phase objective. For under agarose cAMP chemotaxis assay, two troughs were cut after setting of the agarose; one was filled with cells and the other with 4 μM cAMP. 2 mM caffeine was included to prevent cell aggregation. For micropipette chemotaxis assay, differentiated cells were seeded in coverslip chamber filled with DB and allowed to adhere for 15–20 min. A micropipette filled with 1 μM cAMP was placed into the field of view using a micromanipulator. Cell movement was recorded at 15 s intervals using a 40 ×/0.95 oil-immersion objective. To quantify migration parameters, cells were tracked using manual tracking plugin of FIJI ImageJ (https://fiji.sc/) and analyzed using Ibidi chemotaxis tool software.

### Protein Purification

*Escherichia coli* BL21 cells transformed with GST-Teep1^N380^, GST- Teep1^N380M^, or GST-Pten were grown until absorbance at 600 nm of 0.8 and induced with 0.4 mM Isopropyl β-D-1-thiogalactopyranoside (IPTG) for 16–18 h at 20°C. Bacteria pellet was resuspended in ice-cold buffer A (50 mM Hepes pH7.0, 500 mM NaCl, 10% Glycerol, 1 mM PMSF, and 1 mM DTT) and lysed with a high-pressure homogenizer. Cell suspension was centrifuged at 15,000 g for 30 min to pellet the debris. The supernatant was incubated with glutathione sepharose beads (GE Healthcare) for 2 h at 4°C. The protein of interest was eluted with elution buffer (50 mM Hepes pH7.0, 500 mM NaCl, and 10 mM reduced glutathione) followed by gel filtration on a 10/300 G200 Superdex column (GE Healthcare) equilibrated with buffer B (50 mM Hepes pH7.0, 300 mM NaCl, and 1 mM DTT). The GST tag was removed by PreScission protease digestion on the column. The cleaved protein was collected and applied to gel filtration column for further purification. Fractions from the gel filtration column were pooled and concentrated.

### Lipid Dot Blot Assay and Liposome Flotation Assay

Lipid strips (P-6001, P-6002, and S-6000) were obtained from Echelon. Dot blot assay using cell lysates was performed as described before (Yang et al., 2021). When the assay was performed using purified proteins, 0.5 mg/ml protein was used to incubate with pre-blocked lipid strips at room temperature for 1 h.

POPC (850457), NBD-PE (810145), PI3P (850150), PI4P (850157), PI5P (850152), PI(3,4)P_2_ (850153), PI(3,5)P_2_ (850154), PI(4,5)P_2_ (850155), and PIP_3_ (850156) were obtained from Avanti Polar Lipids and dissolved in chloroform. POPC, NBD-PE, and variable PIPs were mixed at molar ratio of 97:1:2 (for binding with Teep1) or 89:1:10 (for binding with Pten). Mixed lipids were dried under a flow of nitrogen gas and in SpeedVac for 1–2 h. The lipid films were resuspended in Hepes-NaCl buffer (50 mM Hepes pH7.0 and 150 mM NaCl) to a final concentration of 5 mM and subjected to freeze-thaw cycles 11 times. Unilamellar liposomes were generated via extrusion through a nanopore membrane with a pore size of 100 nm (Avanti Polar Lipids 610005); the process was repeated 11 times. The liposomes were mixed with purified proteins at molar ratio of 1,000:1 in a 50 μl reaction and incubated at 4°C for 1 h with gentle agitation. 30 μl of the protein-liposome mixture was diluted with 100 μl 1.9 M sucrose, placed at the bottom of a centrifugation tube, and overlaid sequentially with 100 μl 1.25 M sucrose and 20 μl Hepes-NaCl buffer. The sucrose gradient samples were centrifuged at 174,000 g for 1 h at 4°C. Five fractions were collected from the top, mixed with SDS loading buffer, and subjected to SDS-PAGE followed by silver staining. Relative binding was calculated as the sum of band intensities of top two fractions divided by the sum of band intensities of all five fractions.

### Lipid Phosphatase Assay

The phosphatase activity of Mtm6 was determined using a Malachite green assay kit (Echelon Biosciences, Inc., Salt Lake City, UT, United States). Cells expressing Mtm6-GFP were starved without cAMP pulses for 3 h. Cells were washed with wash buffer (20 mM Hepes pH7.2 and 150 mM NaCl), resuspended in lysis buffer (20 mM Hepes pH7.2, 150 mM NaCl, 0.5% NP-40, 5% glycerol, 1 mM DTT, and protease inhibitor), and incubated on ice for 5 min. Lysates were centrifuged for 10 min at 4°C. The supernatant was incubated GFP trap beads (Smart-Lifesciences) for 1 h at 4°C. Beads were washed with wash buffer and reaction buffer (20 mM Hepes pH7.2, 150 mM NaCl, 2 mM DTT, 2 mM CaCl_2_, and 5% Glycerol). Beads containing 200 ng Mtm6-GFP were incubated with 3,000 pmol substrate in a 25 µl reaction for 30 min at 22°C. 20 μl supernatant was mixed with 80 μl Malachite Green solution at room temperature for 30 min. Free phosphate released was measured at 620 nm wavelength.

### Immunoprecipitation Assay and Immunoblotting

For immunoprecipitation assays, cells were starved without cAMP pulses for 3 h. Starved cells were lysed in lysis buffer (10 mM NaPi pH 7.2, 100 mM NaCl, 0.5% NP-40, 10% Glycerol, 1 mM NaF, .5 mM Na_3_VO_4_, and protease inhibitor) and incubated for 10 min on ice. Lysates were centrifuged for 10 min at 4°C. The supernatants were incubated with GFP-Trap beads for 1 h at 4°C. Beads were washed with lysis buffer. Sample were eluted with SDS loading buffer and subjected to SDS-PAGE followed by mass spectrometry analysis or immunoblotting. Mass spectrometry analysis and immunoblotting were performed as described before (Cai et al., 2010; Yang et al., 2021). Anti-GFP antibody (Roche 11814460001) and DsRed polyclonal antibody (Takara 632496) were used for immunoblotting.

### Adhesion Assay

6×10^5^ cells were plated in 6-well tissue-culture plate for 8 h. 2 ml fresh medium was added before the plate was placed on an orbital shaker and rotated at 200 rpm for 1 h. Floating and adherent cells were then counted to calculate the percent of adherent cells.

### Statistical Analysis

Statistical analysis was performed using GraphPad Prism. Statistical significance was determined by unpaired t test or one-way ANOVA with Dunnett or Tukey post-test. In all figures, *** indicates *p* < .001, **p < .01, *p < .05, ns not significant.

## Data Availability

The original contributions presented in the study are included in the article/Supplementary Material, further inquiries can be directed to the corresponding author.
