# Supplementary material for: Gradients of PI(4,5)P2 and PI(3,5)P2 Jointly Participate in Shaping the Back State of Dictyostelium Cells
Source: Front Cell Dev Biol. 2022 Feb 4;10:835185. doi: 10.3389/fcell.2022.835185 (PMC8855053; doi:10.3389/fcell.2022.835185)

Supplemental Information

Legends for supplemental figures

Figure S1. Screen of PH domain-containing proteins. (A) List of PH domain-containing proteins and their localization in vegetative cells. (B) Selected images showing the localization of representative PH domain-containing proteins. Scale bar, 5 μm.

Figure S2. Teep1 localization in comparison with other back proteins. (A) Left: Teep1-GFP translocation in response to folic acid stimulation (folic acid was added at time 0). Right: Quantification of Teep1-GFP translocation (mean ± SEM). Data was from three independent experiments. (B) Left: Diagram of the microfluidic chamber used to generate a cAMP gradient. Right: Localization of Teep1-RFP and PHcrac-GFP in LatA-treated cells in response to cAMP gradient. (C) Time-lapse imaging of vegetative cells expressing Teep1-RFP and PHcrac-GFP. (D-F) Time-lapse imaging of vegetative cells expressing GFP-tagged Pten (D), a tandem PH domain of CynA (E), and PhdB (F). In all images, the arrows point to macropinocytic cups and the asterisks mark newly enclosed macropinosomes. (G) Co-expression of Teep1-RFP and Pten-GFP in vegetative cells. (H) Localization of Teep1-GFP in *pten*$^{-}$ cells and Pten-GFP in *teep1*$^{-}$ cells. Scale bar, 5 μm.

Figure S3. **The N-terminal fragment of Teep1 determines its trailing-edge localization**. (A) Translocation of Teep1^{N411}-GFP in response to cAMP stimulation (1 μM cAMP was added at time 0). (B) Quantification of Teep1^{N411}-GFP translocation in response to cAMP stimulation (mean ± SEM). Data was from three independent experiments. (C) Sequence alignment of the PH domains of Teep1 with the PH domain of PEPP1 reveals conserved charged residues (highlighted by the red boxes) within the β1 and β2 loops. Blue and orange underlines indicate non-canonical and canonical PIP binding sites, respectively. (D) Left: Phyre2 prediction of the structure of the N-terminus of Teep1 (11-247 amino acids). The surfaces are colored according to the vacuum

electrostatics analyzed by Pymol (red to violet represents negative to positive charge). Right: Predicted secondary structure. The four conserved positively charged amino acids are highlighted. (E) Localization of Teep1^{N411}-GFP and Teep1^{N411}-GFP containing point mutations in the PH domains. (F) Left: Localization of Teep1^{N411}-GFP containing the indicated point mutations in WT, *pikI*$^{-}$, or *pikfyve*$^{-}$ cells. Right: Box plot of the membrane-to-cytosol fluorescent intensity ratios of mutated Teep1^{N411}-GFP in the indicated cell lines. Data was from at least two independent experiments. Scale bar, 5 μm.

Figure S4. Generation of *Dd5P4*$^{-}$ and *pikfyve*$^{-}$ cells**.** (A) Design of *Dd5P4* knockout construct. Two independent knockout clones were confirmed by PCR using specific primer pairs indicated by the arrows. (B) *Dd5P4*$^{-}$ cells exhibit severe defects in macropinocytosis, which are rescued by expression of GFP-Dd5P4. Images were acquired after 15 min incubation with TRITC-Dextran. (C) Localization of LimEΔcoil-GFP and TAPP1-GFP in WT and *Dd5P4*$^{-}$ cells. The arrows point to actin comet structures observed in *Dd5P4*$^{-}$ cells. (D) Design of *pikfyve* knockout construct. Two independent knockout clones were confirmed by PCR using specific primer pairs indicated by the arrows. (E) *pikfyve*$^{-}$ cells exhibit enlarged vesicles when placed in low osmolarity developmental buffer. The phenotype is rescued by expression of PIKfyve-GFP. (F) Colocalization of PIKfyve-GFP and RFP-Rab7A. Scale bar, 5 μm.

Figure S5. **PI(3,4)P$_2$ and PI(4,5)P$_2$ in regulating the localization of Teep1.** (A-C) Lipid dot blot assays using cell lysates expressing Teep1-GFP or PHcrac-GFP. (D) Coomassie brilliant blue staining showing purified Teep1^{N380}. (E) Liposome flotation assay with Teep1^{N380}. Five fractions were collected from the top of a sucrose gradient and analyzed by silver staining. (F) Coomassie brilliant blue staining showing purified Teep1^{N380M}, which contains mutations in K11, K158, and R174. (G) Top: Liposome flotation assay with Teep1^{N380M}. Bottom: Quantification of PIP binding of Teep1^{N380M} by liposome flotation assay (mean ± SD). Data was from two independent experiments. (H)

Coomassie brilliant blue staining showing purified Pten. (I) Liposome flotation assay with Pten. (J) Quantification of PIP binding of Pten by liposome flotation assay (mean ± SD). Data was from two independent experiments. (K) Top: Schematic of the chimeric sensor composed of two PX domains fused with Nodulin. Bottom: Localization of GFP-tagged Nodulin, PX-PX, and chimeric sensor in vegetative cells. Arrows point to macropinocytic cups which are devoid of the chimeric sensor. Scale bar, 5 μm.

Figure S6. Generation and characterization of *mtm6*⁻ cells. (A) $PI(3,5)P_2$ in cells is mainly generated by PIKfyve and degraded by Fig4 and myotubularin family of phosphatases. The *Dictyostelium* genome encodes nine putative myotubularin proteins. PTP, protein tyrosine phosphatase domain; PH, pleckstrin homology domain; PX, Phox homology domain; TM, transmembrane domain; ANK, ankyrin repeat. (B) Mtm6-GFP translocation in differentiated WT cells in response to cAMP stimulation (1 μM cAMP was added at time 0). (C) Design of *mtm6* knockout construct. Arrows mark positions of the primers used in PCR and restriction enzyme sites used for digestion of genomic DNA in Southern blot analysis. (D-E) Two independent knockout clones were confirmed by PCR (D) and Southern blot (E). Scale bar, 5 μm.

Figure S7. Generation and characterization of *teep1*⁻ cells. (A) Design of *teep1* knockout construct. (B) Two independent knockout clones were confirmed by Southern blot analysis. Arrows mark restriction enzyme sites used for digestion of genomic DNA. (C) WT and *teep1*⁻ cells were plated as a monolayer on non-nutrient agar to induce development. Typical fields of view were photographed at the indicated time points. Scale bar = 1 mm. (D) Localization of GFP-myosin II in randomly migrating WT and *teep1*⁻ cells. (E) Teep1-GFP was immunoprecipitated from cell lysates and bound proteins were analyzed via mass spectrometry. TalB was identified as a binding partner of Teep1. The table shows the number of identified unique peptides in TalB. (F) Teep1-RFP, but not Leep1-RFP, co-immunoprecipitates with GFP-TalB. (G) Expression of Teep1-RFP, but not Leep1-RFP, recruits GFP-TalB to the cell periphery in LatA treated

cells. (H) Vegetative cells were subjected to a rotational adhesion assay. WT and *teep1*$^{-}$ cells exhibit comparable adhesion. Data was collected from three independent experiments. Scale bar, 5 µm.

Figure S8. Simultaneous depletion of PI(4,5)P_2 and PI(3,5)P_2 causes increased blebbing. (A) Fractions of each migratory mode in WT or *pikfyve*$^{-}$ cells after Inp54p recruitment. Data was collected from at least three independent experiments. (B-C) Time-lapse imaging of Inp54/*pikfyve*$^{-}$ cells (B) and Inp54/*pikfyve*$^{-}$ cells expressing Teep1-GFP (C) before and after the addition of rapamycin. After Inp54 recruitment, cells exhibited increased blebbing (arrow heads). Rapamycin was added at time 0. Scale bar, 5 µm.

Legends for supplemental videos

Video S1. Localization of Teep1-GFP in randomly migrating vegetative cells. Corresponds to Figure 1A. Images were captured at 15 sec per frame (spf) and played back at 6 frames per second (fps). Scale bar, 5 µm.

Video S2. Localization of Teep1-GFP in differentiated WT cells chemotaxing toward cAMP released by a micropipette. Corresponds to Figure 1F. Images were captured at 15 spf and played back at 6 fps. Scale bar, 5 µm.

Video S3. Translocation of Teep1-GFP in response to cAMP stimulation (added at 0:42). Corresponds to Figure 1G. Images were captured at 6 spf and played back at 6 fps. Scale bar, 5 µm.

Video S4. Teep1-GFP translocation in response to cAMP stimulation (added at time 0) in the presence of LatA. Corresponds to Figure 1H. Images were captured at 6 spf and played back at 3 fps. Scale bar, 5 µm.

Video S5. Localization of Teep1-GFP and mCherry-FRB-Inp54p in LatA-treated WT cells before and after the addition of rapamycin (added at 02:15). Corresponds to Figure 4E. Images were captured at 15 spf and played back at 10 fps. Scale bar, 5 µm.

Video S6. Localization of Teep1-GFP and mCherry-FRB-Inp54p in LatA-treated *pikfyve*$^{-}$

cells before and after the addition of rapamycin (added at 02:15). Corresponds to Figure 4F. Images were captured at 15 spf and played back at 10 fps. Scale bar, 5 μm.

Video S7. Localization of Mtm6-GFP in WT cells during macropinocytosis. Corresponds to Figure 5A. Images were captured at 6 spf and played back at 12 fps. Scale bar, 5 μm.

Video S8. Random migration of WT and *teep1*$^{-}$ cells. Corresponds to Figure 6A. Images were captured at 15 spf and played back at 20 fps. Scale bar, 10 μm.

Video S9. mCherry-FRB-Inp54p recruitment in *pikfyve*$^{-}$ cells causes increased blebbing (rapamycin added at 04:15). Corresponds to Figure S8B. Images were captured at 15 spf and played back at 5 fps. Scale bar, 5 μm.

Supplementary Figure S1

A

Gene ID	Localization	Gene ID	Localization
DDB_G0282287	Cytosol	DDB_G0291996	Leading edge enriched
DDB_G0289859	Cytosol	DDB_G0282475	Leading edge enriched
DDB_G0289979	Cytosol	DDB_G0278147	Leading edge enriched
DDB_G0280015	Cytosol	DDB_G0275085	Weak leading edge
DDB_G0288529	Cytosol	DDB_G0290873	Weak leading edge
DDB_G0268704	Cytosol	DDB_G0277777	Lagging edge enriched*
DDB_G0271086	Cytosol	DDB_G0275337	Lagging edge enriched
DDB_G0268384	Cytosol	DDB_G0272372	Lagging edge enriched
DDB_G0293184	Cytosol	DDB_G0288731	Lagging edge enriched
DDB_G0282271	Cytosol	DDB_G0275795	Vesicles*
DDB_G0293266	Cytosol	DDB_G0293644	Vesicles
DDB_G0288377	Cytosol	DDB_G0271552	Vesicles
DDB_G0290023	Cytosol	DDB_G0283415	Vesicles
DDB_G0293928	Cytosol	DDB_G0285303	Vesicles
DDB_G0267854	Cytosol	DDB_G0285859	PM and CV*
DDB_G0290493	Cytosol	DDB_G0291085	PM and CV
DDB_G0278703	Cytosol	DDB_G0293124	PM and puncta
DDB_G0278461	Leading edge enriched*	DDB_G0279123	PM*
DDB_G0268316	Leading edge enriched	DDB_G0282717	PM
DDB_G0291007	Leading edge enriched	DDB_G0293978	PM
DDB_G0277131	Leading edge enriched	DDB_G0278417	PM
DDB_G0291840	Leading edge enriched	DDB_G0293302	Microtubules*
DDB_G0271694	Leading edge enriched	DDB_G0293340	Mitochondria*
DDB_G0292746	Leading edge enriched	DDB_G0273193	ER*
DDB_G0274889	Leading edge enriched	DDB_G0293396	Nucleus

B

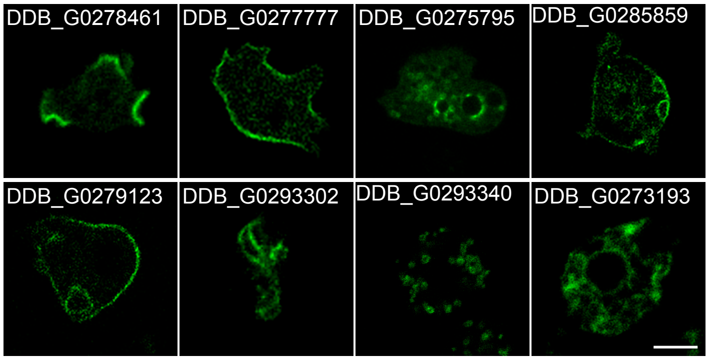

Supplementary Figure S2

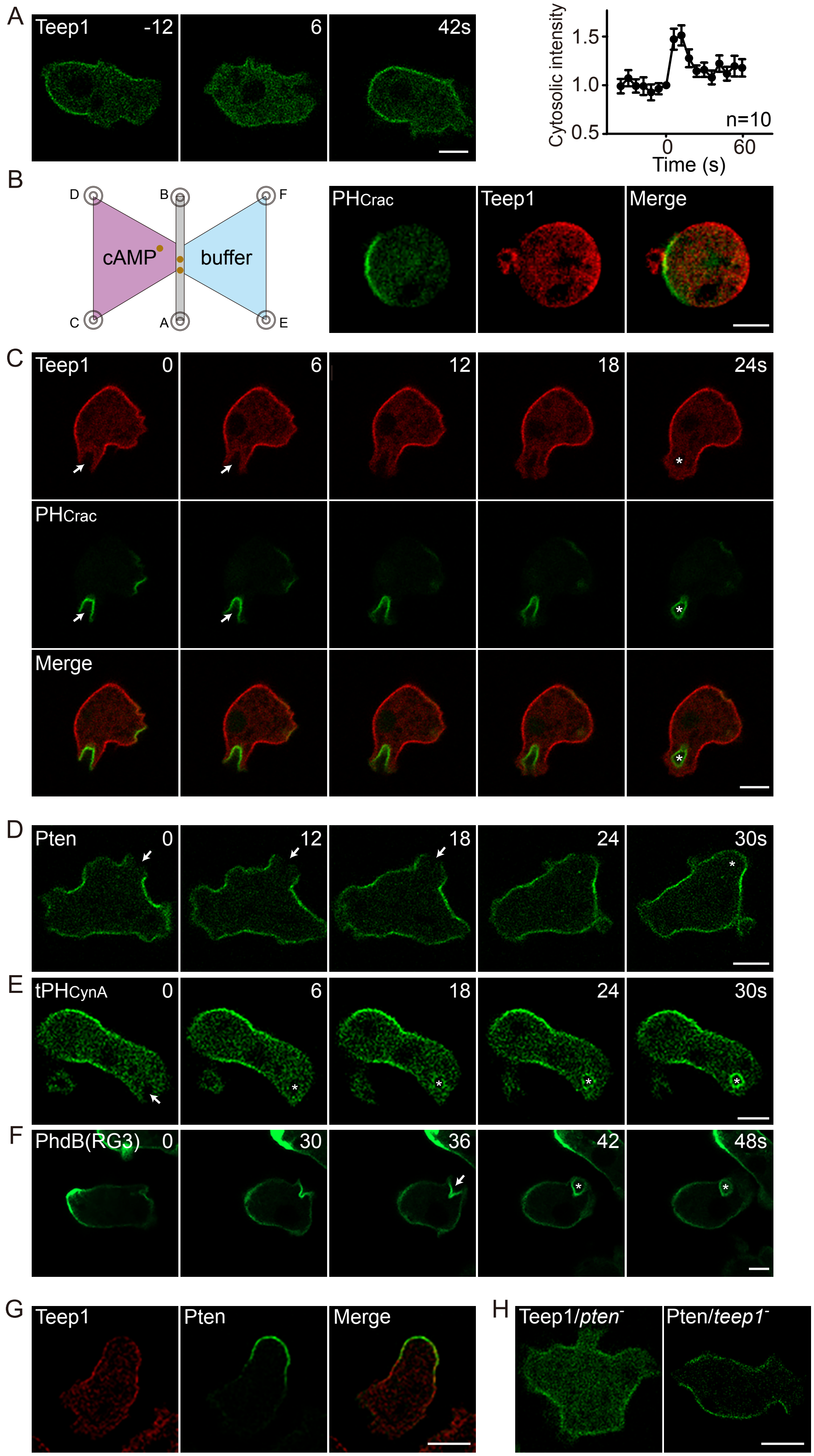

Supplementary Figure S3

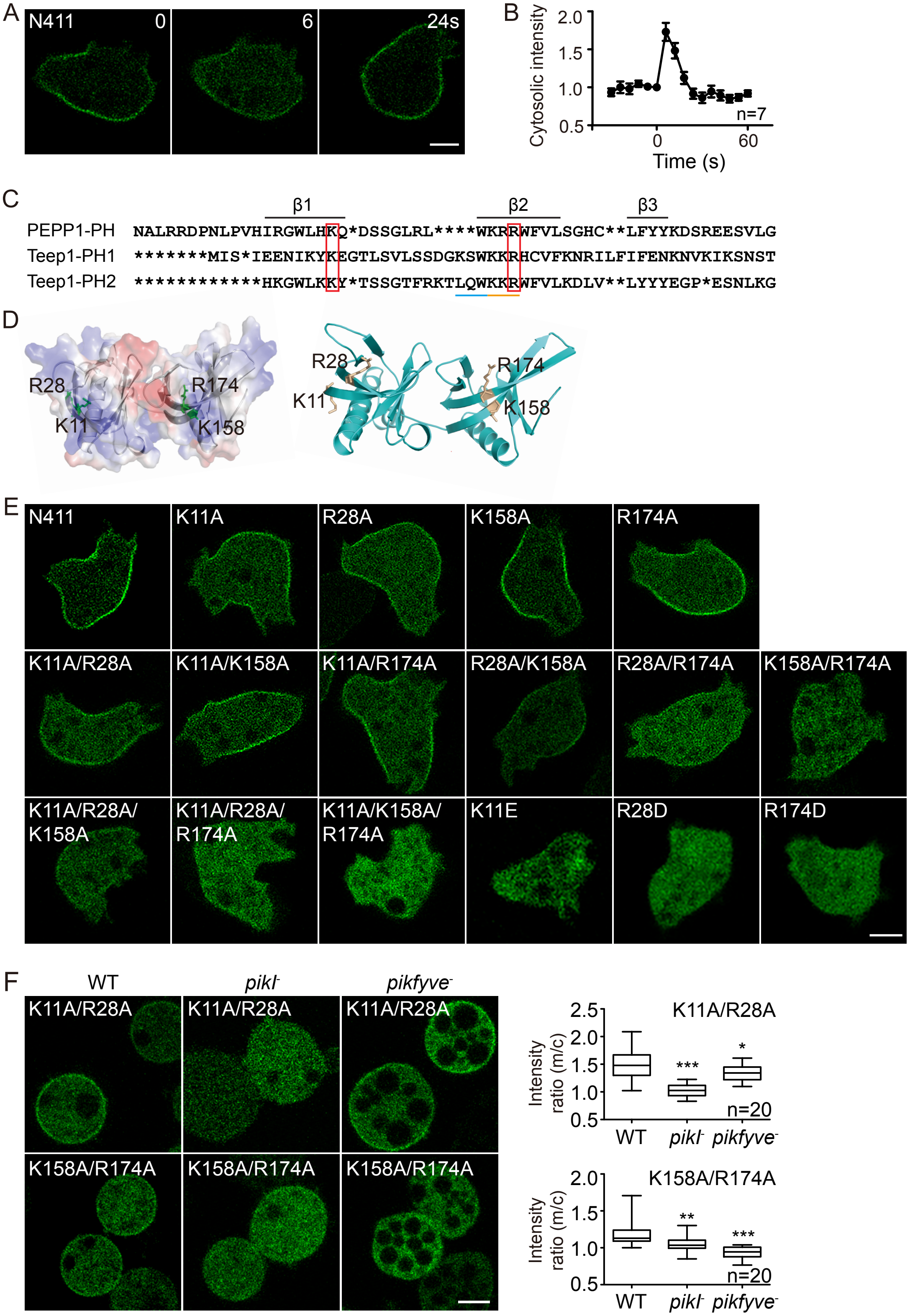

Supplementary Figure S4

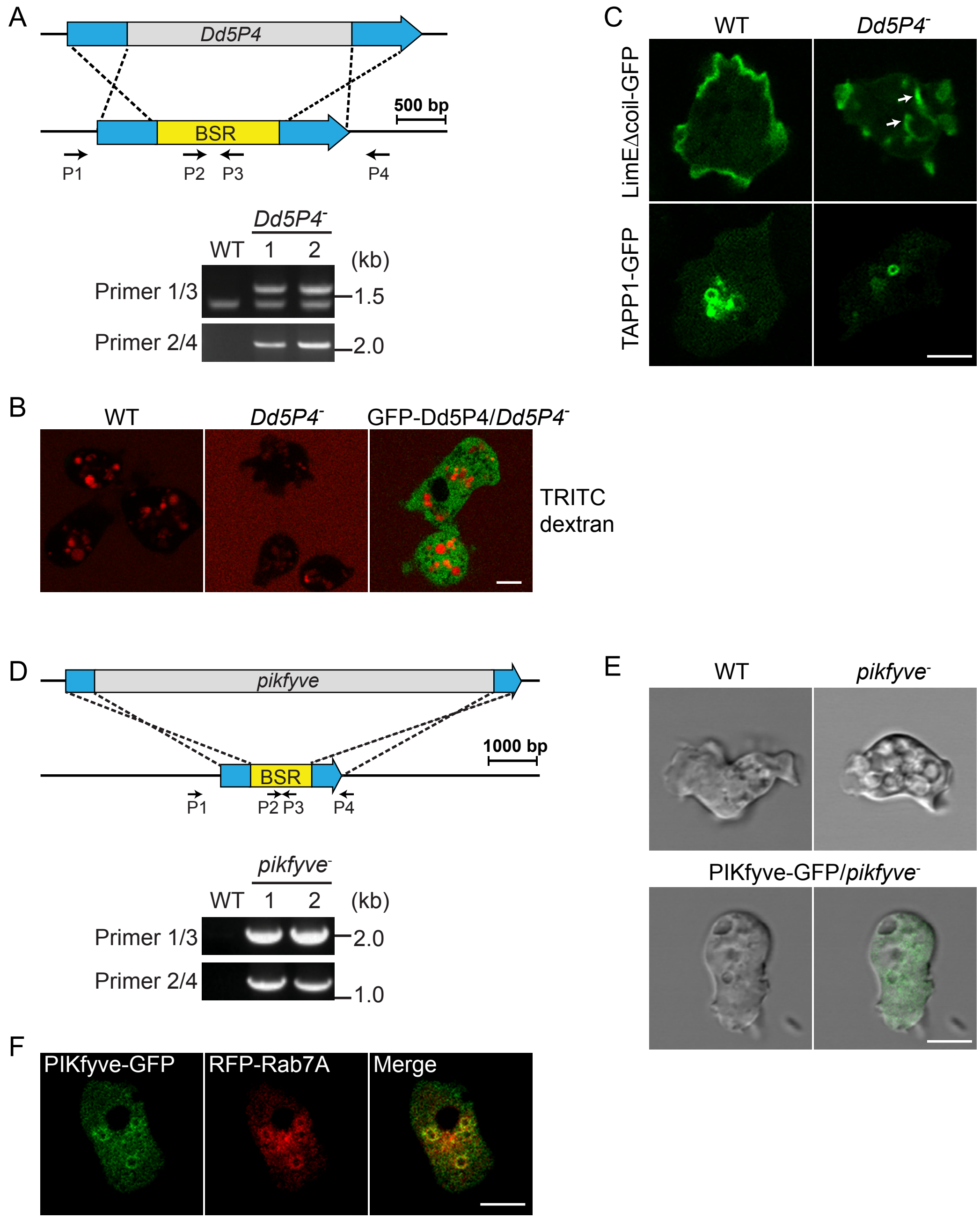

Supplementary Figure S5

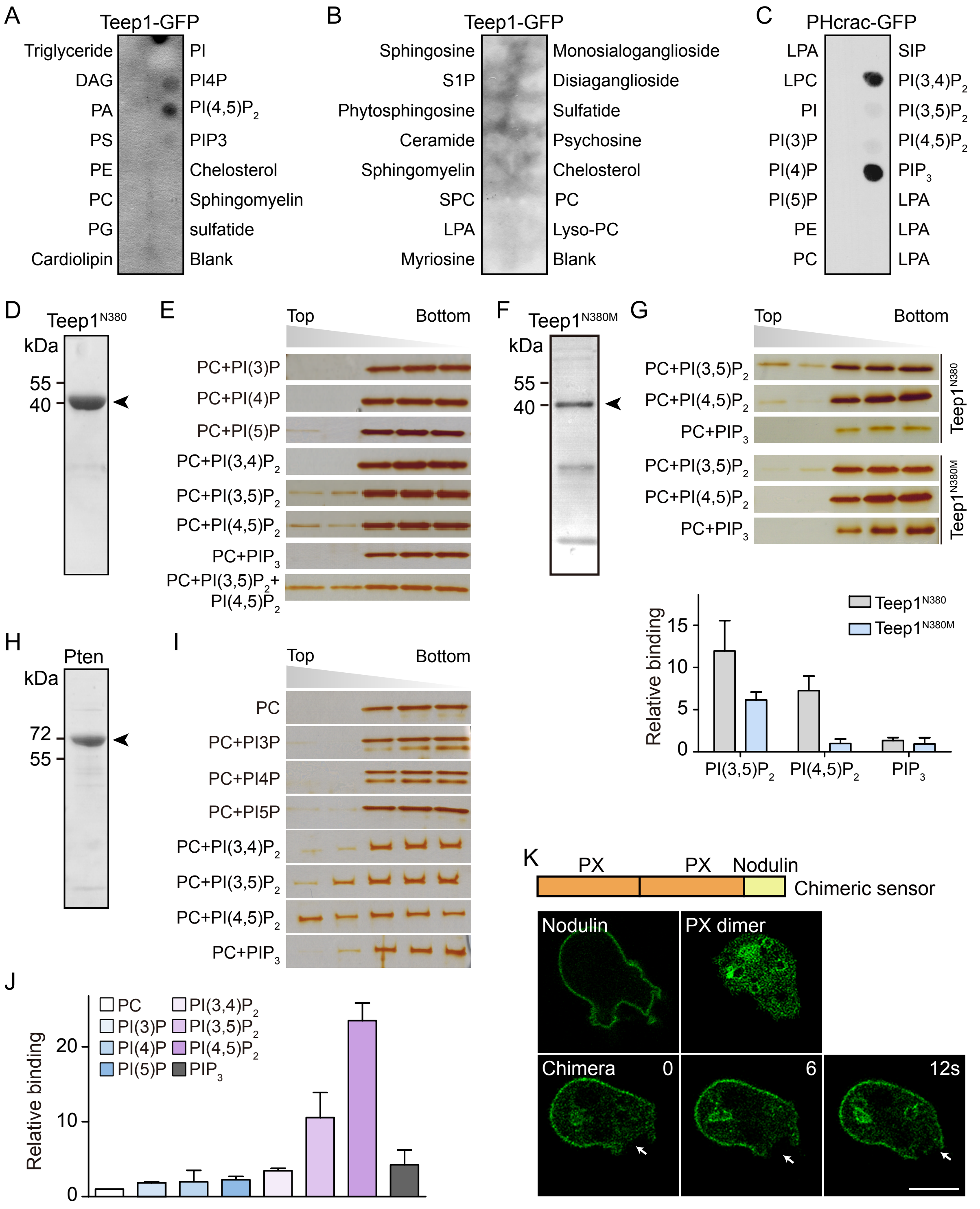

Supplementary Figure S6

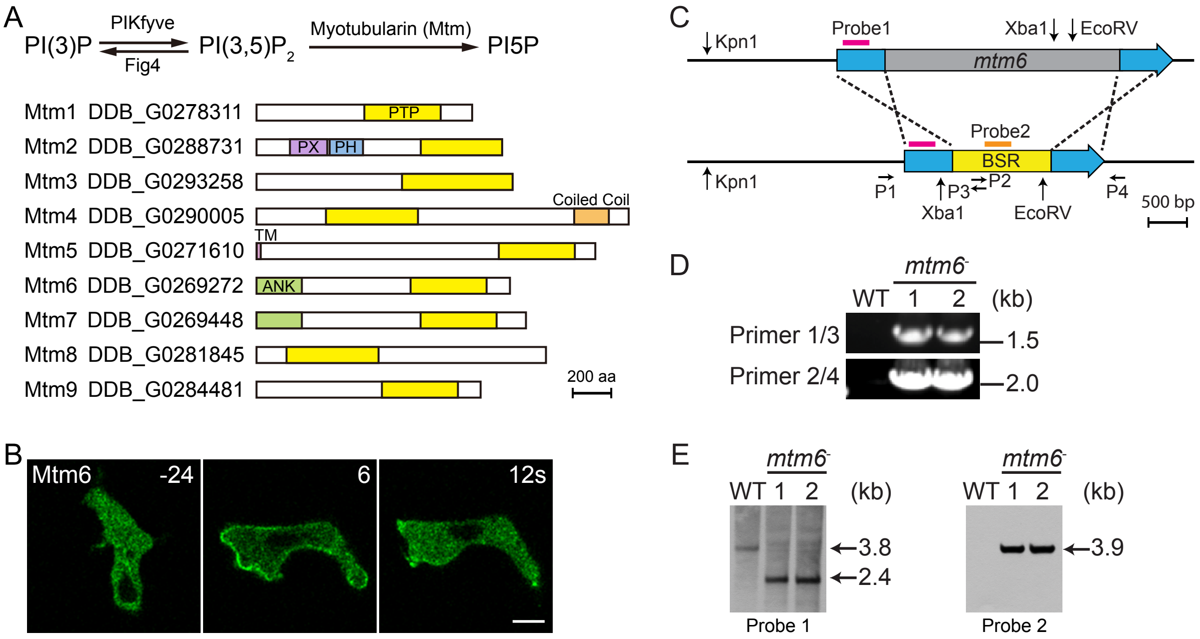

Supplementary Figure S7

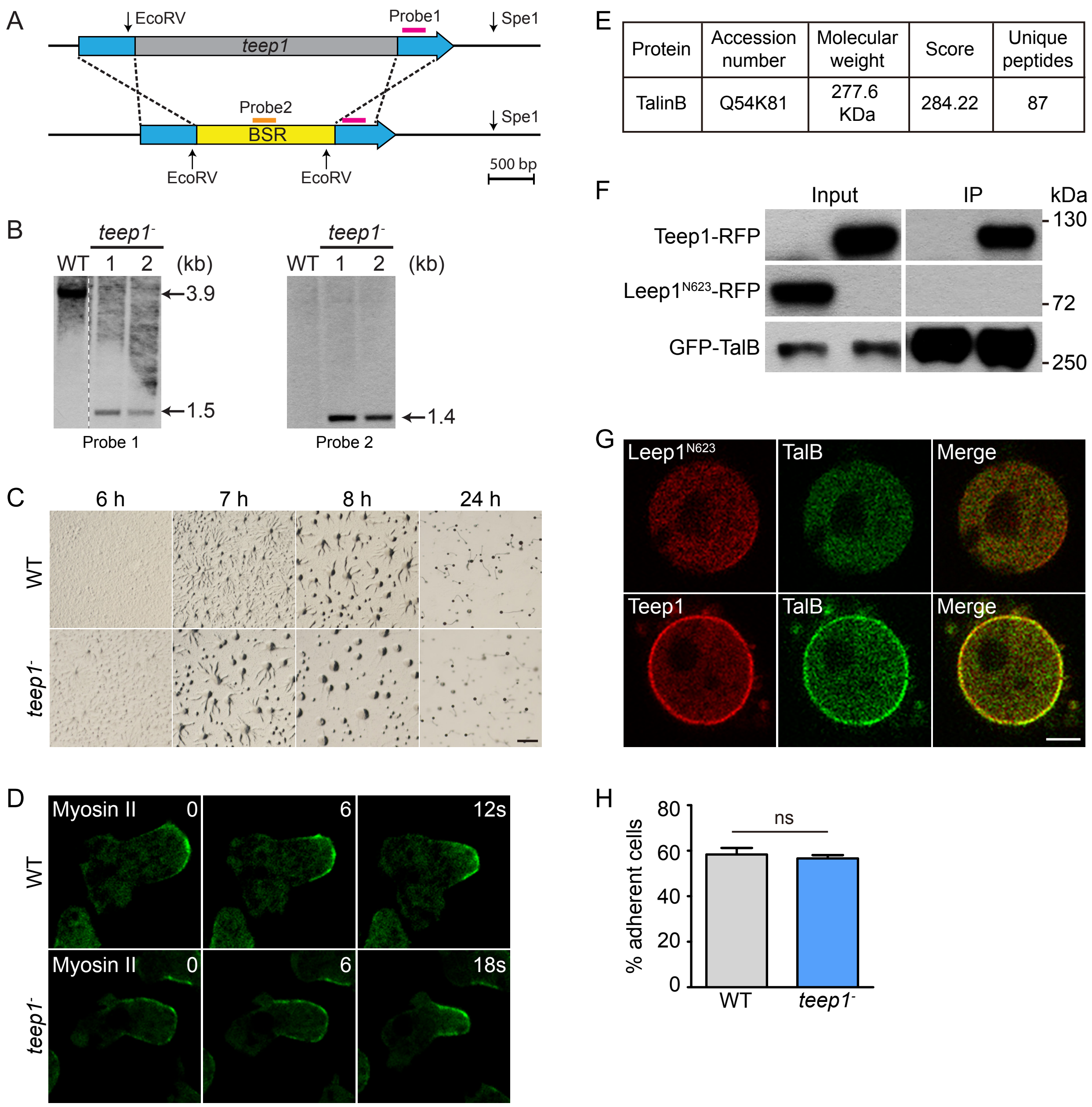

Protein	Accession number	Molecular weight	Score	Unique peptides
TalinB	Q54K81	277.6 KDa	284.22	87

Supplementary Figure S8

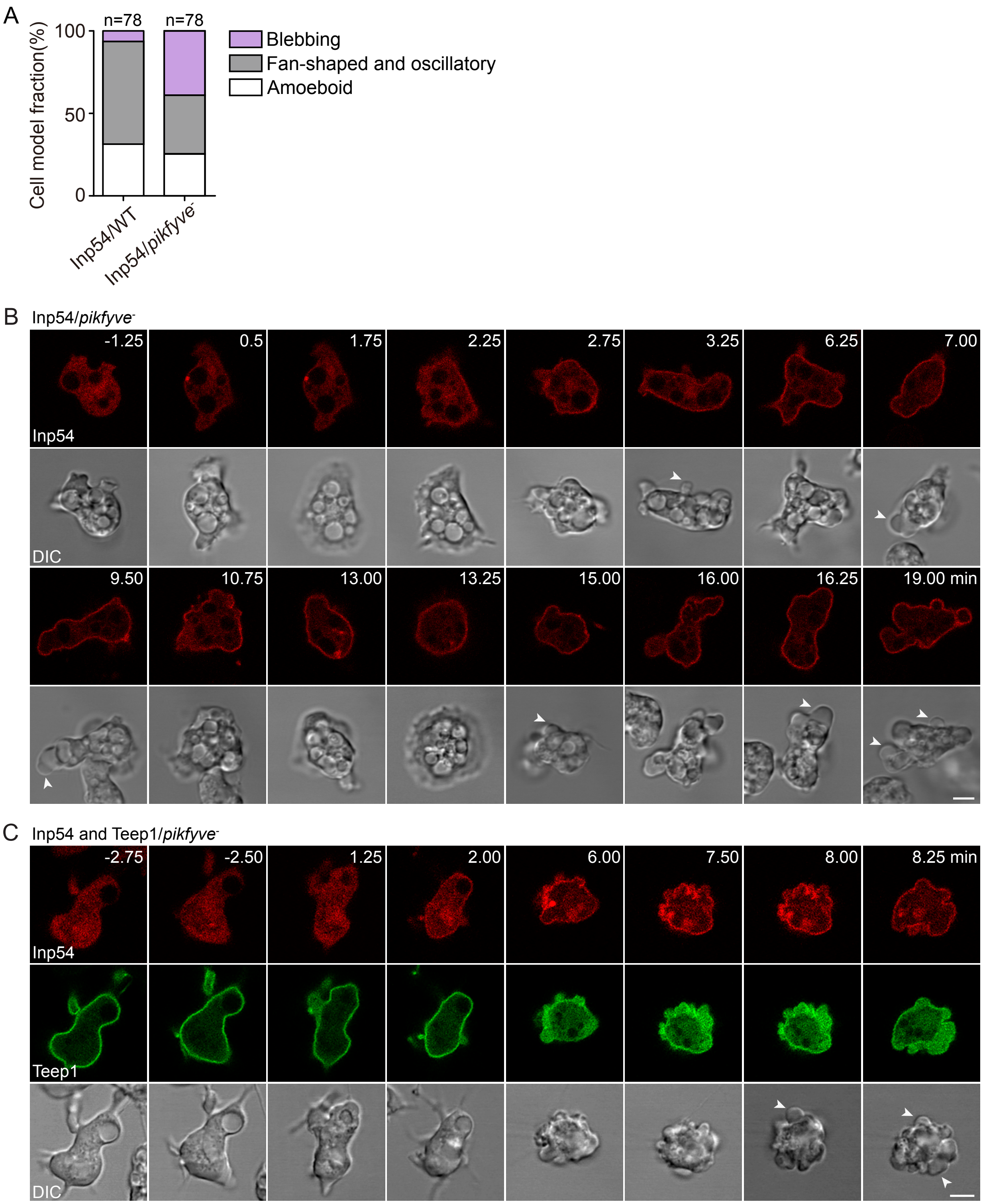

Supplement: Supplementary file 3 [file DataSheet1.pdf]
